# A small molecule targeting CHI3L1 inhibits lung metastasis by blocking IL‐13Rα2‐mediated JNK‐AP‐1 signals

**DOI:** 10.1002/1878-0261.13138

**Published:** 2021-11-24

**Authors:** Yong Sun Lee, Ji Eun Yu, Ki Cheon Kim, Dong Hun Lee, Dong Ju Son, Hee Pom Lee, Jae‐kyung Jung, Nam Du Kim, Young Wan Ham, Jaesuk Yun, Sang‐Bae Han, Jin Tae Hong

**Affiliations:** ^1^ College of Pharmacy & Medical Research Center Chungbuk National University Cheongju Korea; ^2^ Voronoibio Inc. Incheon Korea; ^3^ Department of Chemistry Utah Valley University Orem UT USA

**Keywords:** antitumor therapy, CHI3L1, IL‐13Rα2, K284

## Abstract

Our previous big data analyses showed a high level of association between chitinase 3 like1 (CHI3L1) expression and lung tumor development. In the present study, we investigated whether a CHI3L1‐inhibiting chemical, 2‐({3‐[2‐(1‐cyclohexen‐1‐yl)ethyl]‐6,7‐dimethoxy‐4‐oxo‐3,4‐dihydro‐2‐quinazolinyl}sulfanyl)‐N‐(4‐ethylphenyl)butanamide (K284), could inhibit lung metastasis and studied its mechanism of action. We investigated the antitumor effect of K284 both *in vitro* and *in vivo*. K284 (0.5 mg·kg^−1^ body weight) significantly inhibited lung metastasis in *in vivo* models after injection of murine melanoma cells (B16F10) or adenocarcinomic human alveolar basal epithelial cells (A549). K284 significantly and concentration‐dependently also inhibited cancer cell proliferation and migration in the A549 and H460 lung cancer cell lines. We found that the binding of K284 to the chitin‐binding domain (CBD) of CHI3L1 prevented the binding of CHI3L1 to its receptor, interleukin‐13 receptor subunit alpha‐2 (IL‐13Rα2), thereby suppressing the CHI3L1 signal. This blocking of the CHI3L1‐IL‐13Rα2 signal caused the inhibition of c‐Jun N‐terminal kinase (JNK)‐activator protein 1 (AP‐1) signals, resulting in the prevention of lung metastasis and cancer cell growth. Our data demonstrate that K284 may serve as a potential candidate anticancer compound targeting CHI3L1.

## Graphical abstract

High serum levels of CHI3L1 correlate with poor prognosis and survival in various human carcinomas, including lung cancer. We demonstrated that the selective CHI3L1 inhibitor K284 strongly inhibits lung cancer cell growth and tumor metastasis. K284 inhibited CHI3L1‐IL‐13Rα2 signaling and its downstream pathways. This study indicates K284 as a potential anticancer drug candidate that targets CHI3L1 to block IL‐13Rα2‐mediated JNK‐AP‐1 signaling.

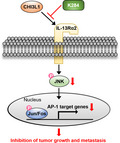

**Abbreviations**
AP‐1: activator protein 1
CBD: chitin‐binding domain
CHI3L1: chitinase 3 like1
JNK: c‐Jun N‐terminal kinases
K284: 2‐({3‐[2‐(1‐Cyclohexen‐1‐yl)ethyl]‐6,7‐dimethoxy‐4‐oxo‐3,4‐dihydro‐2‐quinazolinyl}sulfanyl)‐N‐(4‐ethylphenyl)butanamide

## 1 Introduction

Chitinase 3 like1 (CHI3L1), an 18‐glycosyl hydrolase (GH 18) gene family member, has properties of cytokines and growth factors and is secreted by macrophages, neutrophils, synoviocytes, chondrocytes, cancer cells, epithelial cells, and smooth muscle cells [1, 2, 3]. Its expression is enhanced by several cytokines, including IL‐13, IL‐6, IL‐1β, and IFN‐γ [3, 4]. Abnormal CHI3L1 expression has been implicated in numerous diseases [2, 5]. Increased expression and serum levels of CHI3L1 have been associated with nonmalignant diseases, such as diabetes, Alzheimer's disease, and asthma, as well as with many human neoplasias, including breast, colon, prostate, ovarian, brain, thyroid, bladder, stomach, endometrial, esophageal, pancreatic, head and neck, lung, and liver cancers [1, 4, 5, 6]. High CHI3L1 expression is reported to be independently associated with poorer overall survival in both non‐small‐cell lung carcinoma (NSCLC) and small‐cell lung carcinoma (SCLC) patients [7], and serum CHI3L1 levels in 120 SCLC patients were higher in the group with poorer response to chemotherapy [8]. Moreover, our previous study involving big data analysis and experiments with shRNA of CHI3L1 showed that CHI3L1 is associated with the development of several cancers, especially lung cancer [9].

CHI3L1 stimulates the binding of IL‐13 to IL‐13Rα2, which consequently activates its downstream signaling pathways such as ERK, Akt, and Wnt/β‐catenin signaling pathways [10]. Additionally, the expression of CHI3L1 and IL‐13Rα2 was elevated in many cancers [11]. The role of IL‐13Rα2 in tumorigenesis is debatable as high levels of IL‐13Rα2 were found to inhibit the progression of breast and pancreatic tumors [12]; conversely, IL‐13Rα2 overexpression was found to increase the invasiveness and metastatic potential of ovarian and pancreatic cancer cells [13, 14]. IL‐13Rα2 overexpression induced by treatment with IL‐13 increased the expression of genes responsible for cancer growth and migration such as *ERK*, *AP‐1*, and *MMP* [13, 14]. Despite extensive evidence for the role of CHI3L1 and IL‐13Rα carcinogenesis, studies of compounds preventing CHI3L1/13Rα signaling and consequently inhibiting cancer growth are limited. We screened 1.4 million compounds at ChemBridge and selected 11 compounds after application to the Glide software, which allowed us to use computational docking to predict the binding abilities of the 11 chemical compounds with CHI3L1 [15]. Among the 11 compounds, K284 was selected as the candidate compound since it showed the strongest binding to CHI3L1 and the most significant cytotoxic effects against several cancer cell lines. Thus, in the present study, we investigated whether K284 inhibits lung tumor metastasis through disruption of the CHI3L1/IL‐13Rα2 axis.

## 2 Materials and methods

### 2.1 Chemical compounds

Chemical compounds were purchased from ChemDiv Inc. (San Diego, CA, USA). 11 chemical compounds were named as follows: N‐{[5‐{[2‐(3,4‐Dihydro‐1(2H)‐quinolinyl)‐2‐oxoethyl]sulfanyl}‐4‐(3‐methylphenyl)‐4H‐1,2,4‐triazol‐3‐yl]methyl}‐2‐furamide (C592‐3733), N‐(2‐Methoxyphenyl)‐2‐{[2‐(4‐methoxyphenyl)‐5H‐chromeno[2,3‐d]pyrimidin‐4‐yl]sulfanyl}acetamide (C683‐0269), N‐(2‐{[3‐Bromo‐1‐(4‐methylbenzyl)‐1H‐1,2,4‐triazol‐5‐yl]sulfanyl}phenyl)‐3‐fluorobenzamide (F938‐0173), N‐Allyl‐2‐[(6‐butyl‐1,3‐dimethyl‐2,4‐dioxo‐1,2,3,4‐tetrahydropyrido[2,3‐d]pyrimidin‐5‐yl)sulfanyl]acetamide (G721‐0282), 2‐({3‐[2‐(1‐Cyclohexen‐1‐yl)ethyl]‐6,7‐dimethoxy‐4‐oxo‐3,4‐dihydro‐2‐quinazolinyl}sulfanyl)‐N‐(4‐ethylphenyl)butanamide (K284‐6111), 2‐[(3‐Cyclopropyl‐4‐oxo‐3,4‐dihydrothieno[3,2‐d]pyrimidin‐2‐yl)sulfanyl]‐N‐[2‐(4‐sulfamoylphenyl)ethyl]acetamide (K292‐1690), 7‐Hydroxy‐3‐(1‐methyl‐1H‐benzimidazol‐2‐yl)‐8‐[(2‐methyl‐1‐piperidinyl)methyl]‐2H‐chromen‐2‐one (3570‐0063), N‐[3‐(2,3‐Dihydro‐1H‐indol‐1‐yl)‐3‐oxopropyl]‐2,5‐dimethyl‐4‐(1‐pyrrolidinylcarbonyl)‐1H‐pyrrole‐3‐sulfonamide (F535‐0062), 2‐{[2‐Ethyl‐7‐oxo‐6‐(2‐phenylethyl)‐6,7‐dihydro‐2H‐pyrazolo[4,3‐d]pyrimidin‐5‐yl] sulfanyl}acetamide (G020‐0160), 2‐[(6‐Ethyl‐1,3‐dimethyl‐2,4‐dioxo‐1,2,3,4‐tetrahydropyrido[2,3‐d]pyrimidin‐5‐yl)sulfanyl]acetamide (G857‐0938), and N‐(2‐{[3‐Chloro‐1‐(4‐fluorobenzyl)‐1H‐1,2,4‐triazol‐5‐yl]sulfanyl} phenyl)‐2,6‐difluorobenzamide (L150‐0261).

### 2.2 Lung and melanoma metastasis models

C57BL/6 mice (male) and BALB/c nude mice (male) were purchased from DBL (Eumseong, Korea). They were provided standard mice feed and water *ad libitum*. For melanoma metastasis, B16F10 mouse melanomas were injected intravenously (4 × 10^4^ cells/100 μL in phosphate‐buffered saline (PBS)) to 8‐week‐old C57BL/6 mice. The next day, 0.5 mg per kg of K284 was injected through intravenous injection and these injections were performed every 3 days for 3 weeks. For human lung cancer metastasis, A549 cells were injected intravenously (1 × 10^7^ cells/100 μL in PBS) to 8‐week‐old BALB/c nude mice. The next day, 0.5 mg·kg^−1^ of K284 was injected through intravenous injection and these injections were performed every 3 days for 8 weeks. All protocols were reviewed and approved by the Chungbuk National University Institutional Animal Care and Use Committee (IACUC) and complied with the Korean National Institute of Health Guide for the Care and Use of Laboratory Animals (CBNUA‐1073‐17‐01).

### 2.3 Cell culture

B16F10 mouse melanoma, LLC mouse Lewis lung carcinoma, and A549 and H460 NSCLC cells were obtained from the American Type Culture Collection (Manassas, VA, USA). A549 and H460 cells were cultured in RPMI 1640 medium supplemented with 10% heat‐inactivated fetal bovine serum (FBS), 100 U·mL^−1^ penicillin, and 100 μg·mL^−1^ streptomycin. B16F10 and LLC cells were cultured in DMEM supplemented with 10% FBS, 100 U·mL^−1^ penicillin, and 100 μg·mL^−1^ streptomycin. Cell cultures were maintained in an incubator with a humidified atmosphere of 5% CO_2_ at 37 °C.

### 2.4 Cell viability assay

Cell viability assay was performed as described previously [16]. A549, H460, and LLC cell lines were seeded in 96‐well plates. On the next day, the cells were treated with K284 (0–5 μm) for 24 h after which the cell viability was measured by MTT [3‐(4,5‐dimethylthiazol‐2‐yl)‐2,5‐diphenyltetrazolium bromide] assay (Sigma‐Aldrich, St. Louis, MO, USA) according to the manufacturer’s instructions. Briefly, MTT (5 mg·mL^−1^) was added and plates were incubated at 37 °C for 4 h before dimethyl sulfoxide (100 μL) was added to each well. Finally, the absorbance in each well was read at a wavelength of 540 nm using a plate reader.

### 2.5 Western blotting

Western blotting analysis was performed as described previously [9]. The membranes were immunoblotted with specific primary antibodies. Antibodies were purchased from Cell Signaling (Danvers, MA, USA; caspase‐3, p53, p‐ERK, ERK, p‐p38, p38, and p‐JNK), Abcam (Cambridge, UK; CHI3L1, PCNA, cyclin D1, MMP‐2, MMP‐9, and MMP‐13), Abclonal (Wuhan, China; p‐AKT and MMP‐3), Abnova (Taipei, Taiwan; p‐c‐fos), GeneTex (San Antonio, TX, USA; p‐c‐jun, IL‐13Rα1, and IL‐13Rα2), and Santa Cruz Biotech (Dallas, TX, USA; Bax, Bcl‐2, Cdk2, Cdk4, Cdk6, cyclin E, c‐Jun N‐terminal kinases (JNK), AKT, c‐jun, c‐fos, p21, and β‐actin). Reactions were detected using secondary antibodies and visualized by using a chemiluminescence detection system.

### 2.6 Hematoxylin and eosin staining and Immunohistochemistry (IHC)

Lung and tumor tissues were dissected and immediately fixed in 4% formaldehyde solution and embedded in paraffin. The tissues were then sectioned into 10‐μm‐thick slices. Hematoxylin and eosin staining and IHC were performed as described previously [9]. For IHC, the melanoma metastasis and lung tumor tissue sections were blocked and incubated with antibodies for CHI3L1, IL‐13Rα1, IL‐13Rα2, Cyclin D1, MMP‐9, PCNA, and cleaved caspase‐3 at the appropriate dilution (1 : 100 dilution). A negative control was performed, by omitting the primary antibody.

### 2.7 Immunofluorescence staining

A549 and H460 cells were plated in a poly‐l‐lysine‐coated 8‐chamber slide (Corning Falcon; Corning, NY, USA), and then, cells were transfected with FL or Δ278‐294 CHI3L1 expression vector. After 24 h, cells were treated with K284 (5 μm). Cells were fixed with methanol. Cells were blocked and stained with c‐jun antibody for 2 h. The slide was stained with an anti‐rabbit secondary antibody labeled with Alexa Fluor 568 (1 : 400 dilution; Molecular Probes Inc., Eugene, OR, USA). DAPI was used for counterstaining.

### 2.8 Luciferase assay

Cell viability assay was performed modifying the method described previously [17]. A549 and H460 cells were plated in 12‐well plates (1 × 10^5^ cells·well^−1^) and transiently transfected with AP‐1 plasmid (Stratagene, La Jolla, CA, USA) or Myc‐Chi3L1 plasmid using a mixture of plasmid and Lipofectamine 3000 in OPTI‐MEM according to the manufacturer’s specifications (Invitrogen, Carlsbad, CA, USA) for 24 h. The transfected cells were treated with recombinant human IL‐13 (10 ng·mL^−1^) for another 24 h. Luciferase activity was measured by using a luciferase assay kit (Promega, Madison, WI, USA) and a luminometer as described by the manufacturer’s specifications (WinGlow, Bad Wildbad, Germany).

### 2.9 Flow cytometry

Flow cytometry was performed as described previously [16]. A549 and H460 cells (7 × 10^5^ cells) were transferred into 60‐mm dishes and incubated in a 37 °C incubator (5% CO_2_) until the cells returned to normal conditions. The cells were treated with K284 for 24 h. Then, both adherent and floating were harvested using trypsin/EDTA 0.25%. Harvested cells were transferred into a tube and centrifuged. Pelleted cells were washed with cold PBS and resuspended in PBS containing propidium iodide (PI) (Sigma‐Aldrich) (40 μg·mL^−1^), RNase (100 μg·mL^−1^; Gibco, Grand Island, NY, USA), and Triton X‐100 (Sigma‐Aldrich) at 37 °C for 10 min. The samples were analyzed using fluorescence‐activated cell sorting. The percentages of cells in G1, S, and G2/M phases were calculated using Cell Quest.

### 2.10 Detection of apoptosis

TdT‐mediated dUTP‐biotin nick end‐labeling (TUNEL) assay was performed as described previously [16]. A549 and H460 cells were cultured on 8‐chamber slides and then treated with K284 (0–5 μm) for 24 h. The cells were washed twice with phosphate‐buffered saline (PBS) and fixed by incubation in 4% paraformaldehyde in PBS for 1 h at room temperature. TUNEL assays were performed using the DeadEnd™ Fluorometric TUNEL System (Promega, Madison, WI, USA) according to the manufacturer’s instructions. The total number of cells in a given area was determined using DAPI staining. The apoptotic index was determined as the number of TUNEL‐positive stained cells divided by the total cell number × 100%.

### 2.11 Electrophoretic mobility shift analysis (EMSA)

FL or Δ274‐294 human CHI3L1 expression vector‐transfected A549 and H460 cells were treated with K284. EMSA was used for testing *in vitro* DNA‐binding activity of AP‐1 according to the manufacturer's recommendations (Promega, Madison, WI, USA).

### 2.12 Docking experiment

A docking experiment was performed as described previously [16]. A docking study of CHI3L1 and K284 was conducted using AutoDock Vina. Only one monomer of the homodimeric CHI3L1 crystal structure (PDB code: 1HJX) was used in the docking experiments and conditioned using AutoDockTools by adding all polar hydrogen atoms. Initially, the grid box was centered on the CHI3L1 monomer and its size was adjusted to include the whole monomer. After the initial search of the binding site, docking experiments were performed again using a smaller grid size (30 × 30 × 30). Docking experiments were performed at various exhaustiveness values of the default: 16, 24, 32, 40, and 60. Molecular graphics for the best binding model was generated using discovery studio visualizer 2.0 (Dassault Systemes, San Diego, CA, USA).

### 2.13 Pull‐down assay

Pull‐down assay was conducted as described previously [18]. K284 was conjugated with epoxy‐activated Sepharose 6B. K284 (1 mg) was dissolved in 1 mL of coupling buffer (0.1 M NaHCO_3,_ pH 11.0 containing 0.5 M NaCl). The proteins were resolved by SDS/PAGE followed by immunoblotting with antibody against CHI3L1 (1 : 1000 dilutions; Abcam).

### 2.14 GST pull‐down assay

GST pull‐down assay was performed as described previously [16]. For expression of the CHI3L1 deletion mutant, plasmids harboring full‐length wild‐type (pcDNA‐FLAG‐full‐length CHI3L1) and mutant (T456A/T456F) CHI3L1 were transfected into A549 cells using the TnT Quick Coupled Transcription/Translation System (Promega). GST fusion proteins were collected on glutathione‐Sepharose beads (Amersham Pharmacia Biotech, Piscataway, NJ, USA), incubated at 4 °C for 4 h with 200 µg of cell lysate or boiled with 2.5× sample buffer for 3 min, and centrifuged. The supernatant fraction was examined by 12% SDS/PAGE. The binding was detected by autoradiography or western blotting.

### 2.15 Immunoprecipitation assay

Immunoprecipitation assay was performed as described previously [16]. 6×Myc‐tagged human CHI3L1 or Δ278‐294 CHI3L1 expression vector‐transfected A549 and H460 cells were harvested in NET‐NL lysis buffer containing 50 mm Tris (pH 7.5), 5 mm EDTA, 150 mm NaCl, 1 mm DTT, 0.01% NP‐40, 0.2 mm PMSF, and a mixture of protease inhibitors (Roche, Mannheim, Germany). Cell lysates were clarified by centrifugation before incubating at 4 °C overnight with a monoclonal antibody against 6×Myc or IL‐13Rα2. An aliquot of 50 µL prewashed protein G‐agarose was added, and the incubation was continued for 2 h at 4 °C. Immunoprecipitates were recovered by centrifugation, washed three times in NET‐NW buffer, and resolved by western blotting.

### 2.16 Trans‐well migration assay

Migration of human lung cancer cells, A549 and H460, was quantitatively performed on permeable inserts (8‐μm pore trans‐well; Corning Inc.). K284 (5 μm)‐treated A549 and H460 cells were plated at 2.0 × 10^4^ cells per well and incubated at 37 °C, 5% CO_2_ in a humidified incubator for 17 h. After incubation, the cells were fixed and stained with trypan blue for 20 min. Nonmigrated cells on the inside of the wells were removed with a cotton swab, and the images, captured under a light microscope (Olympus, Tokyo, Japan) at ×200 magnification, were analyzed, using imagej software (National Institutes of Health, Bethesda, MD, USA).

### 2.17 Human samples

Human tissue samples from patients with lung cancer and healthy controls were obtained from the Keimyung University Dongsan Medical Center, Chonnam National University Hospital, and Chonbuk National University Hospital. All studies using human samples were conducted in accordance with the Declaration of Helsinki and were approved by the Ethics Committee of Chungbuk National University Medical Center (Institutional Review Board Approval No. CBNU‐IRB‐2011‐U01). The experiments were undertaken with the understanding and written consent of each subject.

### 2.18 Enzyme‐linked immunosorbent assay (ELISA)

The whole blood was centrifuged at 8000 ***g*** for 10 min, and Chi3L1 levels were measured using Mouse Chi3L1 DuoSet ELISA Kit (R&D system Inc., Minneapolis, MN, USA) according to the manufacturer's specifications.

### 2.19 Statistical analysis

Statistical analysis was performed as described previously [16]. Statistical analysis was carried out with spss version 18.0 (SPSS Inc., Chicago, IL, USA). All error bars reported are the standard deviation (SD) unless otherwise indicated. Pairwise comparisons were made using one‐way Student’s *t*‐test. Multiple comparisons were made using one‐way analysis of variance followed by Tukey’s tests. Differences between groups were considered significant at *P*‐values below 0.05.

## 3 Results

### 3.1 Selective CHI3L1 inhibitor, K284, strongly inhibits lung cancer cell growth

High serum levels of CHI3L1 correlate with poor prognosis and survival in various human carcinomas, including lung cancer [19, 20, 21, 22, 23, 24, 25, 26]. We screened 1.4 million compounds against the ChemBridge library (San Diego, CA, USA) and selected 11 compounds by implementing structure‐based virtual screening (Fig. S1A). We then obtained docking scores using the Glide software (Schrödinger, New York, NY, USA), which allows computational docking analysis to predict the abilities of chemical compounds to bind to CHI3L1 [15]. Among the 11 compounds, K284 showed the strongest binding to CHI3L1 (Fig. S1B). We also tested the inhibitory effects of these selected 11 compounds, which can bind to CHI3L1, on lung cancer cell growth. Similar to the results of binding affinity, among the 11 compounds, K284 showed the most effective and concentration‐dependent cancer cell growth inhibition in lung cancer cell lines (H460 and LLC) and a concentration‐dependent effect in A549 cancer cells (Fig. S2A–C). The transcriptional activities of CHI3L1 using luciferase assay further showed the significant and effective inhibition by K284 in both A549 and H460 cells (Fig. S2D). We also found that IL‐13‐induced CHI3L1 expression was most significantly decreased by K284 (data not shown). We thus selected K284 for further study. We also found that K284 inhibited the growth of various cancer cell lines but less effectively (IC_50_ = 5–10 μm) than in lung cancer cells (Fig. S2E).

### 3.2 K284 inhibits cancer cell growth, arrests cell cycle, and induces apoptotic cell death

We then investigated the concentration‐dependent effects of K284 treatment in human lung cancer cell lines A549 and H460. We found that K284 inhibits the growth of A549 (IC_50_ = 2.5 μm, Fig. S3A) and H460 cells (IC_50_ = 2.7 μm, Fig. S3B) in a concentration‐dependent manner. To further investigate the fate of lung cancer cells, we assessed cell cycle, migration, and apoptosis. K284 caused G0/G1 cell cycle arrest (Fig. S3C,D), inhibited migration (Fig. S3E,F), and induced apoptotic cell death (Fig. S3G,H). However, migration was significantly inhibited (IC_50_ = 0.8 μm in A549; IC_50_ = 1.5 μm in H460). Moreover, the protein expression of PCNA, cyclin D1, cyclin E, Cdk 2, Cdk 4, and Cdk 6 (cell growth and cycle‐related proteins; Fig. S4A,B); MMP‐2, MMP‐3, MMP‐9, and MMP‐13 (migration marker proteins; Fig. S4C,D); and Bcl‐2 and pro‐caspase‐3 was decreased by K284 treatment; however, the expression of Bax, cleaved caspase‐3, p21, and p53 (cell death‐related proteins; Fig. S4E,F) was increased in a concentration‐dependent manner in A549 and H460 cells.

### 3.3 K284 inhibits tumor metastasis

**Fig. 1:**
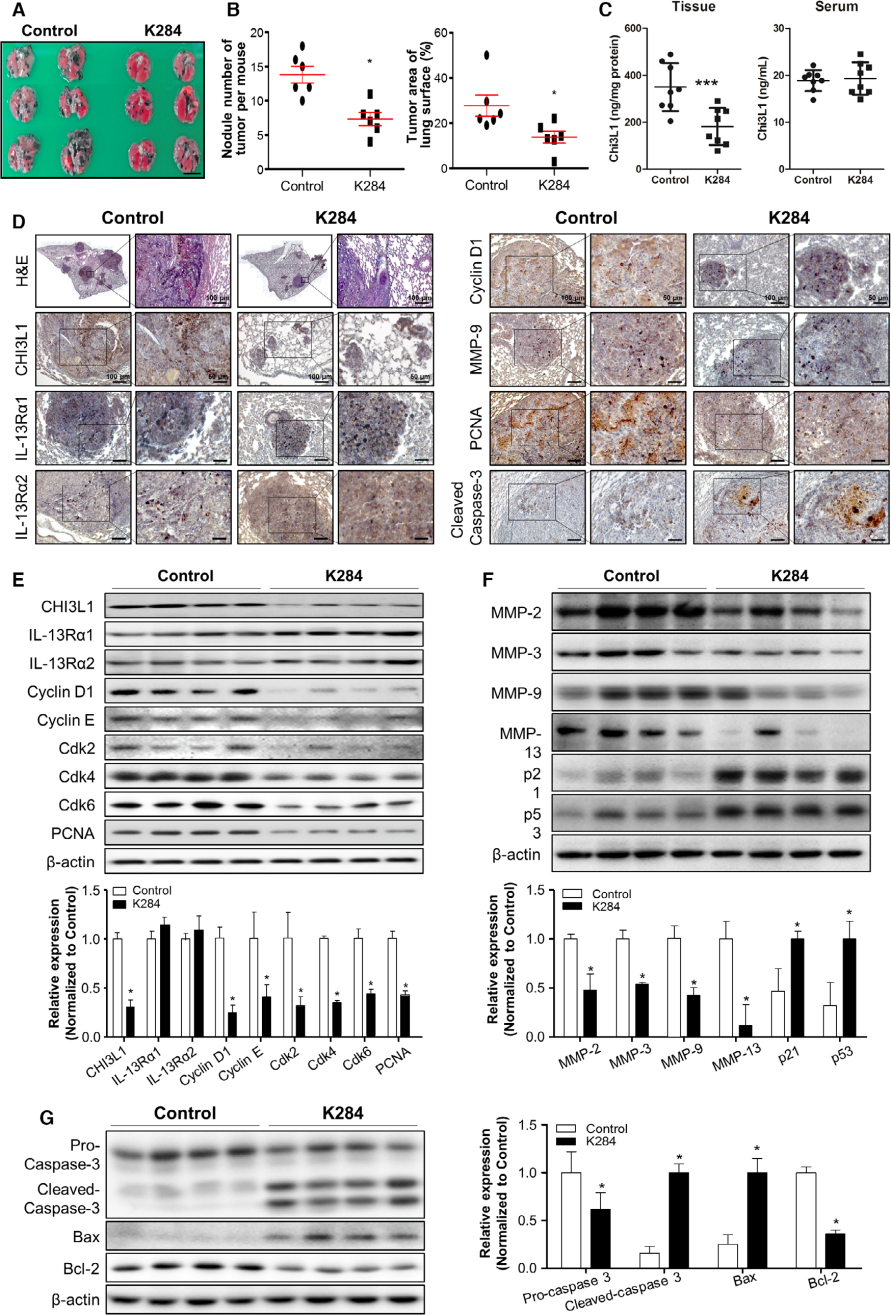
Effect of K284 on B16F10 melanoma metastasis to the lungs in C57BL/6 mice. (A) Images of lung metastasis of melanoma in C57BL/6 mice. B16F10 melanoma cells were injected in C57BL/6 mice that were then intravenously administered via the tail vein saline (left) or 0.5 mg·kg^−1^ K284 (right) at 3‐day intervals for 3 weeks. *n* = 6 per group. Scale bar, 5 mm. (B) The number of tumor nodules and area of mouse lung tissues were measured. *n* = 6 per group. *P < 0.05 (vs. control). (C) The tissue and serum levels of CHI3L1 were analyzed by ELISA. *n* = 6. ***P < 0.001 (vs. control). (D) Hematoxylin and eosin (H&E) staining of lung tissue sections. Immunohistochemical analysis for CHI3L1, IL‐13Rα1, IL‐13Rα2, cyclin D1, MMP‐9, PCNA, and cleaved caspase‐3 in lung tissue sections. Scale bar, 100 μm. Scale bar of enlarged image, 50 μm. (E) Western blot analysis for CHI3L1, IL‐13Rα1, IL‐13Rα2, cyclin D1, cyclin E, Cdk 2, Cdk 4, Cdk 6, and PCNA from the lung tumor metastatic tissue lysate of C57BL/6 mice. The relative protein expression in the K284‐treated group compared with that in the control group is shown in the graphs. *P < 0.05 (vs. Control). (F) Western blot analysis for MMP‐2, MMP‐3, MMP‐9, MMP‐13, p21, and p53 from the lung metastatic tissue lysate. The relative protein expression in the K284‐treated group compared with that in the control group is shown in the graphs. *P < 0.05 (vs. control). (G) Western blot analysis for caspase‐3, Bax, and Bcl‐2 from the lung tumor metastatic tissue lysate of C57BL/6 mice. The relative protein expression in the K284‐treated group compared with that in the control group is shown in the graphs. *P < 0.05 (vs. control). All error bars of graph reported are the standard deviation (SD) from three independent experiments.

**Fig. 2:**
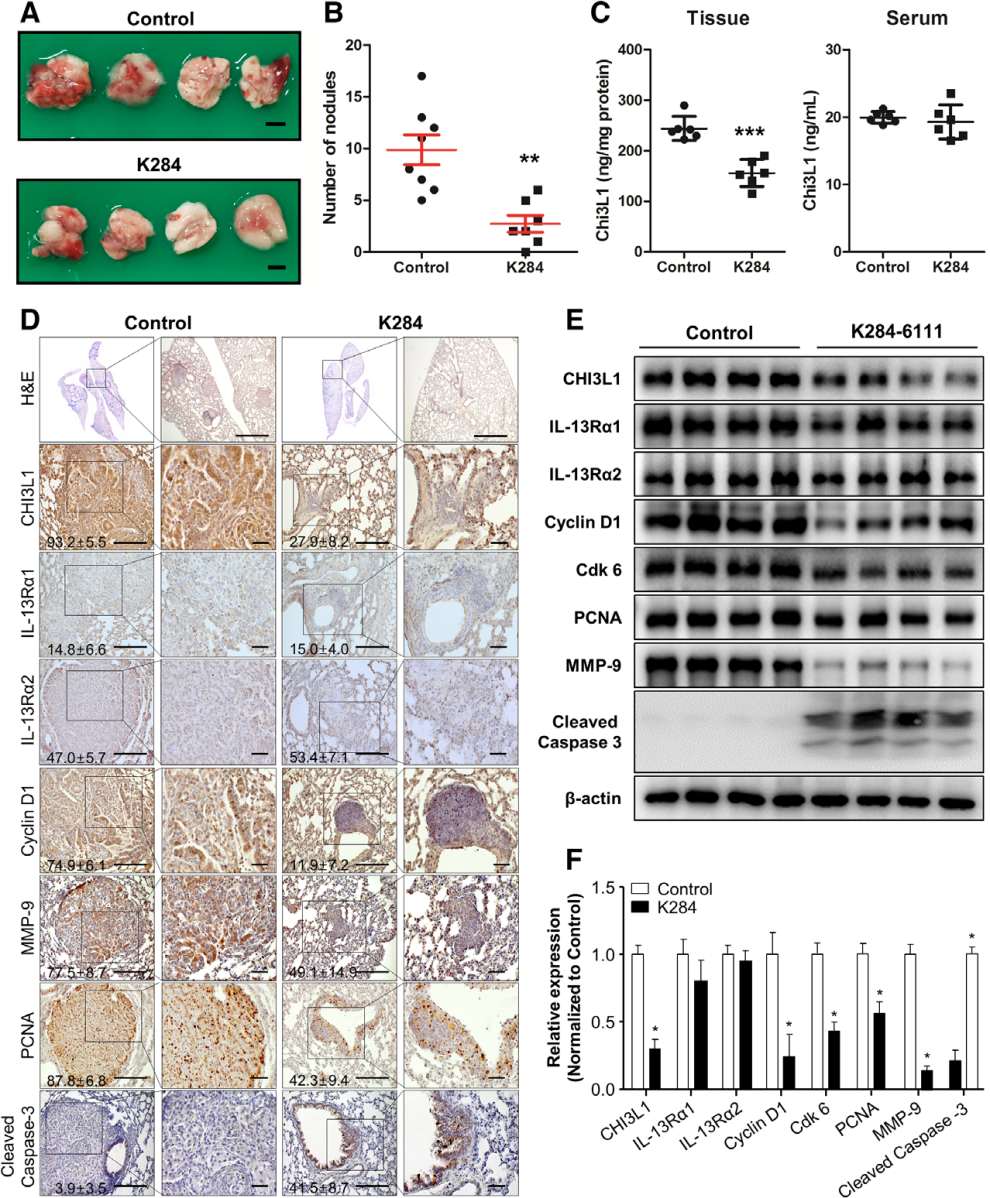
Effect of K284 on the metastasis of A549 cells to the lungs of C57BL/6 mice. (A) Images of lung metastasis of human lung cancer cells in BALB/c nude mice. A549 human lung cancer cells were injected in BALB/c nude mice that were then intravenously administered via the tail vein saline (left) or 0.5 mg·kg^−1^ K284 (right) at 3‐day intervals for 8 weeks. *n* = 8 for the control group; *n* = 7 for the K284‐treated group. Scale bar, 2 mm. (B) The number of tumor nodules in mouse lung tissues was counted. *n* = 6. **P < 0.01 (vs. control). (C) The tissue and serum levels of CHI3L1 were analyzed by ELISA. *n* = 6. ***P < 0.001 (vs. control). (D) H&E staining of lung tissue sections. Immunohistochemical analysis for CHI3L1, IL‐13Rα1, IL‐13Rα2, cyclin D1, MMP‐9, PCNA, and cleaved caspase‐3 in lung tissue sections. The percentage of positive cells in immunohistochemistry staining was calculated and shown in the images. Scale bar, 50 μm. (E, F) Western blot analysis for CHI3L1, IL‐13Rα1, IL‐13Rα2, cyclin D1, Cdk 6, PCNA, MMP‐9, and cleaved caspase‐3 from the lung tumor metastatic tissue lysate. The relative protein expression in the K284‐treated group compared with that in the control group is shown in the graphs. *P < 0.05 vs. control. All error bars of graph reported are the standard deviation (SD) from three independent experiments.

We first investigated the antimetastatic effect of K284 *in vivo*. As shown in Fig. 1A,B, tumor metastasis, number of nodules, and tumor area were decreased in K284‐treated mice. The level of CHI3L1 in B16F10 lung metastasis tissue was significantly decreased but was not much altered in the serum of K284‐treated mice (Fig. 1C). Hematoxylin and eosin (H&E) staining (Fig. 1D) showed a few adenocarcinomas in K284‐treated mice; however, tumors from untreated mice were well‐differentiated lung adenomas. Immunohistochemistry analysis (Fig. 1D,E) showed that the protein expression of CHI3L1, PCNA, cyclin D1, and MMP‐9 was suppressed, but that of cleaved caspase‐3 was increased. However, the expression of IL‐13Rα1 and IL‐13Rα2 was not changed in K284‐treated lung tumor tissues (Fig. 1D,E). Additionally, we analyzed metastasis, cell cycle, proliferation, and apoptotic cell death‐mediated protein expression by western blotting. As shown in Fig. 1F, the protein expression of cell growth‐related and cell cycle‐related proteins was suppressed by the K284 treatment. The expression of migration marker proteins was also suppressed, but that of p21 and p53 was increased in tumor tissues of K284‐treated mice (Fig. 1G). We also found that K284 suppresses the expression of the antiapoptotic protein Bcl‐2 but enhances the expression of the proapoptotic proteins Bax and cleaved caspase‐3 (Fig. 1H). To further investigate the lung metastasis of lung cancer cells, we assessed lung metastasis using A549 cells *in vivo*. As shown in Fig. 2A,B, lung metastasis was suppressed, and nodule numbers and tumor area were decreased in K284‐treated mice. K284 significantly reduced the tissue levels of CHI3L1, but not in serum CHI3L1 (Fig. 2C). Similar to melanoma metastasis of lung tissues, H&E staining showed a few undifferentiated adenocarcinomas in the K284‐treated mice (Fig. 2D). Immunohistochemistry and western blotting analyses (Fig. 2D,E) showed reduced protein expression of CHI3L1, PCNA, cyclin D1, Cdk 6, and MMP‐9, but the expression of cleaved caspase‐3 was increased; however, IL‐13Rα1 and IL‐13Rα2 levels were not changed in K284‐treated lung metastasis tumor tissues (Fig. 2D). These results were associated with our previous findings showing the significance of CHI3L1 in tumor development and indicated that K284 is a useful anticancer drug candidate.

### 3.4 K284 directly binds to the CBD of CHI3L1

**Fig. 3:**
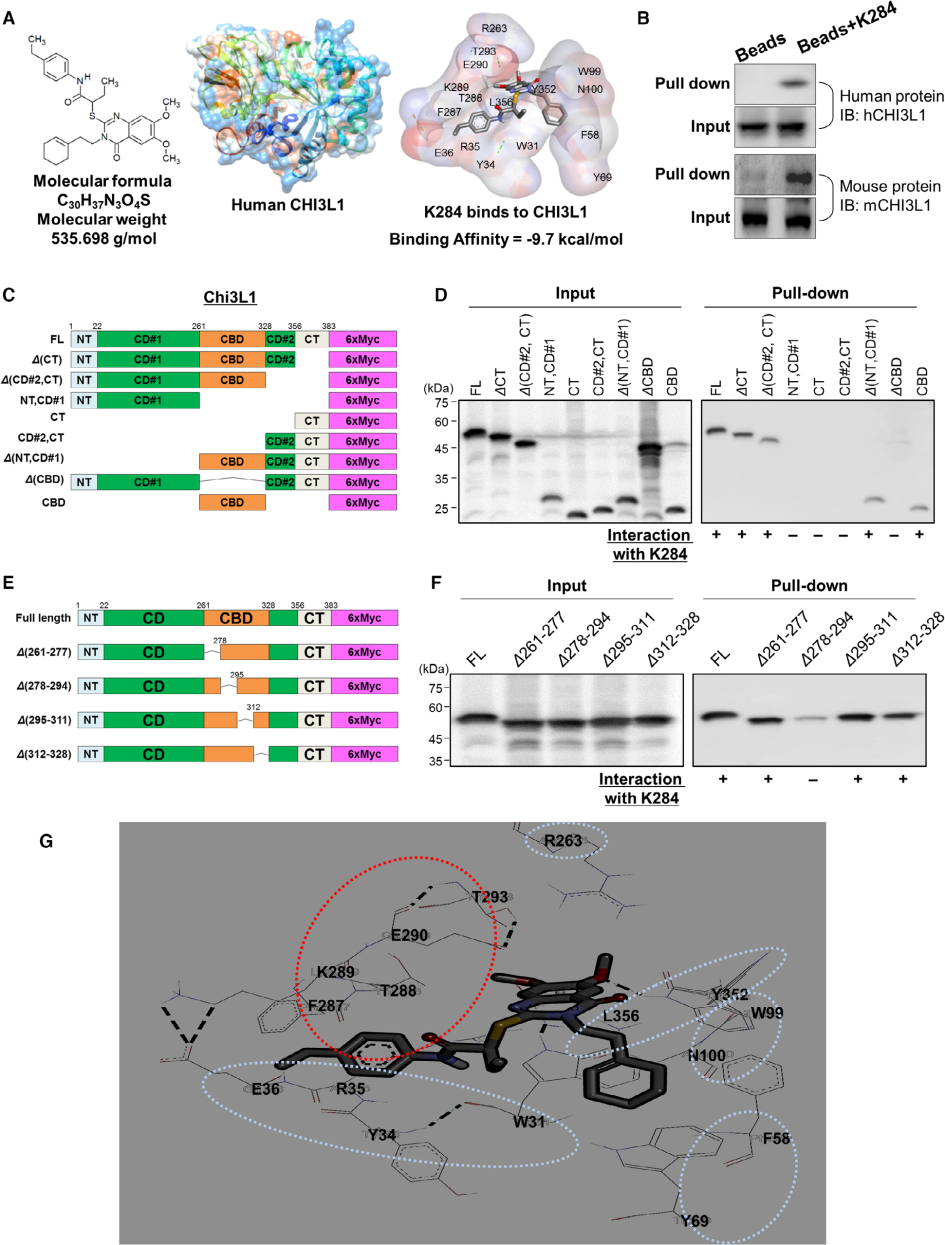
Structure of K284 and molecular binding between K284 and CHI3L1. (A) Chemical structures of K284 (left) and CHI3L1 (middle) and the docking model between K284 and CHI3L1 with the binding affinity (right). (B) Pull‐down assay showing the interaction between K284 and human or mouse CHI3L1. K284 was conjugated with epoxy‐activated Sepharose 6B. (C) Schematic domain structures of 6×Myc‐tagged human CHI3L1 partly deleted mutants. (D) Pull‐down assay showing the interaction between K284 and mutated human CHI3L1. Lane 1, size marker; lane 2, FL CHI3L1‐6×Myc; lane 3, ΔCT CHI3L1‐6×Myc; lane 4, Δ(CD#2 and CT) CHI3L1‐6×Myc; lane 5, Δ(NT and CD#1) CHI3L1‐6×Myc; lane 6, ΔCT CHI3L1‐6×Myc; lane 7, Δ(CD#2 and CT) of CHI3L1‐6×Myc; lane 8, Δ(NT and CD#1) CHI3L1‐6×Myc; lane 9, ΔCBD CHI3L1‐6×Myc; and lane 10, CBD of CHI3L1‐6×Myc. K284 was conjugated with epoxy‐activated Sepharose 6B. Supernatants of immunoprecipitated samples were loaded as ‘Input’, and the precipitated samples were loaded as ‘Pull‐down’. (E) Schematic domain structures of 6×Myc‐tagged human CHI3L1 partly deleted mutants of the chitin‐binding domain. (F) Pull‐down assay showing the interaction between K284 and mutated human CHI3L1. Lane 1, size marker; lane 2, full‐length CHI3L1‐6×Myc; lane 3, Δ261‐277 CHI3L1‐6×Myc; lane 4, Δ278‐294 CHI3L1‐6×Myc; lane 5, Δ295‐311 CHI3L1‐6×Myc; and lane 6, Δ312‐328 CHI3L1‐6×Myc. K284 was conjugated with epoxy‐activated Sepharose 6B. Supernatants of immunoprecipitated samples were loaded as ‘Input’, and the precipitated samples were loaded as ‘Pull‐down’. (G) Docking model of K284 to amino acids of CHI3L1.

We have confirmed that K284 inhibits the growth of the cancer cells and tumor metastasis in in vitro and in vivo experiments. To investigate whether K284 inhibits CHI3L1 signaling in cancer cell growth, we first analyzed the possibility of the physical interaction between CHI3L1 and K284 *in* *silico*. The interaction could change the receptor binding affinity and further suppress downstream signals of CHI3L1. Thus, we investigated the binding of K284 to CHI3L1, and the docking model showed that K284 (Fig. 3A, left) interacts with human CHI3L1 (Fig. 3A, middle) with a strong binding affinity (Fig. 3A, right; *K_d_* = −9.7 kcal·mol^−1^). To assess the binding of K284 to CHI3L1 *in vitro*, we used a pull‐down assay to investigate whether K284 binds to CHI3L1 protein from the cell lysate of both human and mouse cell lines and found that K284 binds with both mouse and human CHI3L1 (Fig. 3B, right). Next, we employed the 6×Myc‐tagged human CHI3L1 protein deletion mapping to identify the regions of CHI3L1 binding to K284. The full length of the CHI3L1 region (FL) has 358 amino acids (AA), and it is classified as having a N‐terminal (NT; 1‐22 AA), a catalytic domain (CD; 23‐356 AA), a chitin‐binding domain (CBD; 262‐328 AA), and a C‐terminal (CT; 357‐383 AA). The structures of expression vector were 1‐383 CHI3L1 (FL), 1‐356 AA (ΔCT), 357‐383 AA (CT), 1‐328 (ΔCD#2, ΔCT), 329‐383 AA (CD#2,CT), 1‐261 AA (NT, CD#1), 262‐383 AA (ΔNT, ΔCD#1), 1‐261 AA and 329‐383 AA (ΔCBD), and 262–328 AA (CBD) (Fig. 3C). As shown in Fig. 3D, K284 interacts with CHI3L1 FL and several other deletion‐mutated CHI3L1; however, the interaction between K284 and CBD‐deleted CHI3L1 was rarely detected (Fig. 3D). These data confirmed that the CBD of CHI3L1 is the binding site for K284. We applied four types of human CHI3L1 protein expression vectors with deletions of 16 AA ranges on CBD to decide the regions where CBD interacts with K284 (Fig. 3E). CHI3L1 CBD mutation sites, such as FL, Δ261‐277, Δ295‐311, and Δ312‐328, showed interaction with K284, but Δ278‐294 CHI3L1 protein showed weak interaction (Fig. 3F). These data indicate that K284 binds to the 278‐294 region of CHI3L1. The result obtained for the pull‐down assay agrees with the result of the *in* *silico* docking prediction model, indicating that phenylalanine 287, threonine 288, lysine 289, glutamate 290, and threonine 293 of CBD of human CHI3L1 bind to K284(Fig. 3G).

### 3.5 Biological effects of the binding of K284 to CHI3L1

**Fig. 4:**
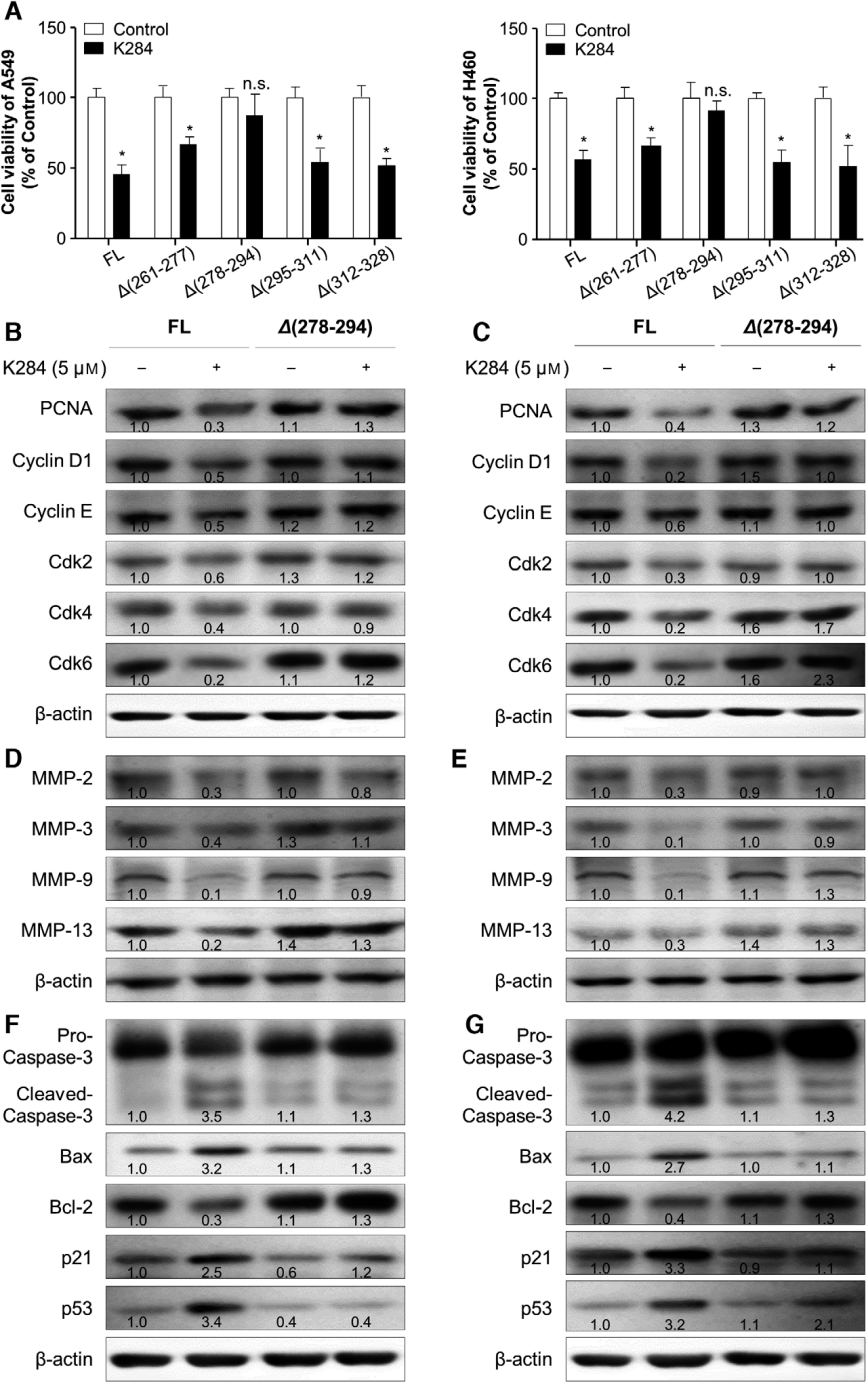
Effects of mutations of the CHI3L1 to K284 on the expression of proteins related to cell proliferation, migration, and apoptosis. (A) The proliferation of A549 and H460 cells with mutated human CHI3L1 expression vectors. Lane 1, FL CHI3L1‐6×Myc; lane 2, Δ261‐277 CHI3L1‐6×Myc; lane 3, Δ278‐294 CHI3L1‐6×Myc; lane 4, Δ295‐311 CHI3L1‐6×Myc; and lane 5, Δ312‐328 CHI3L1‐6×Myc. Transfected cells were treated with or without 5 μm K284 and incubated for 24 h. Cell viability was measured by the MTT assay. *P < 0.05 (vs. control). All error bars of graph reported are the standard deviation (SD) from three independent experiments. (B–G) A549 cells and H460 cells were transfected with FL or Δ278‐294 mutants of human CHI3L1‐6×Myc expression vector and treated with 5 μm of K284 for 24 h. Western blot analysis for cyclin D1, cyclin E, Cdk 2, Cdk 4, Cdk 6, and PCNA from A549 cells (B) and H460 cells (C). Western blot analysis for MMP‐2, MMP‐3, MMP‐9, and MMP‐13 from A549 cells (D) and H460 cells (E). Western blot analysis for caspase‐3, Bax, Bcl‐2, p21, and p53 from A549 cells (F) and H460 cells (G). Western blotting was conducted two times with duplicate samples. The values below the bands represent the band density.

To investigate the binding effect of K284 to CHI3L1, we questioned whether the binding affinity is conversely related to the inhibitory effect of K284 on cancer cell. Thus, we first examined the inhibitory effects of K284 in CBD‐mutated CHI3L1‐transfected cancer cells, by assessing the cell proliferation. We found that the cell proliferation inhibitory effects were eliminated in Δ278‐294 CHI3L1‐transfected A549 and H460 cells (Fig. 4A). We also investigated cell cycle stage, cancer cell migration, and cell proliferation during K284 treatment in either CHI3L1 FL‐ or Δ278‐294 CHI3L1‐transfected A549 and H460 lung cancer cells. G0/G1 phase arrest was observed by K284 treatment in CHI3L1 FL‐transfected cells, but this phenomenon was absent in Δ278‐294 CHI3L1‐transfected A549 and H460 cells (Fig. S5A,B). Cell migration and proliferation were inhibited by K284 treatment in CHI3L1 FL‐transfected cells, whereas cell migration was not inhibited in Δ278‐294 CHI3L1‐transfected A549 and H460 cells (Fig. S5C,D). We also found that the inhibition of cell proliferation was abolished in Δ278‐294‐deleted CHI3L1 (Fig. S5E,F). Next, we investigated whether these biological changes are associated with the inhibition of cell growth, migration, and apoptosis‐related gene expression. The expression of cell growth‐ and cell cycle‐related proteins was suppressed by K284 treatment in CHI3L1 FL‐transfected A549 and H460 cell lines, but these inhibitory effects of K284 were not seen in Δ278‐294 CHI3L1‐transfected A549 and H460 cells (Fig. 4B,C). We also found that the expression of migration marker proteins was decreased by K284 treatment in CHI3L1 FL‐transfected human A549 and H460 cells (Fig. 4D,E). In addition, the expression of cleaved caspase‐3, Bax, p21, and p53 was increased but that of Bcl‐2 and pro‐caspase‐3 was decreased by K284 treatment in CHI3L1 FL‐transfected cells; however, these alterations by K284 treatment were not seen in Δ278‐294 CHI3L1‐transfected A549 and H460 cells (Fig. 4F,G).

### 3.6 K284 inhibits CHI3L1/IL‐13Rα2 signaling and its downstream pathways

**Fig. 5:**
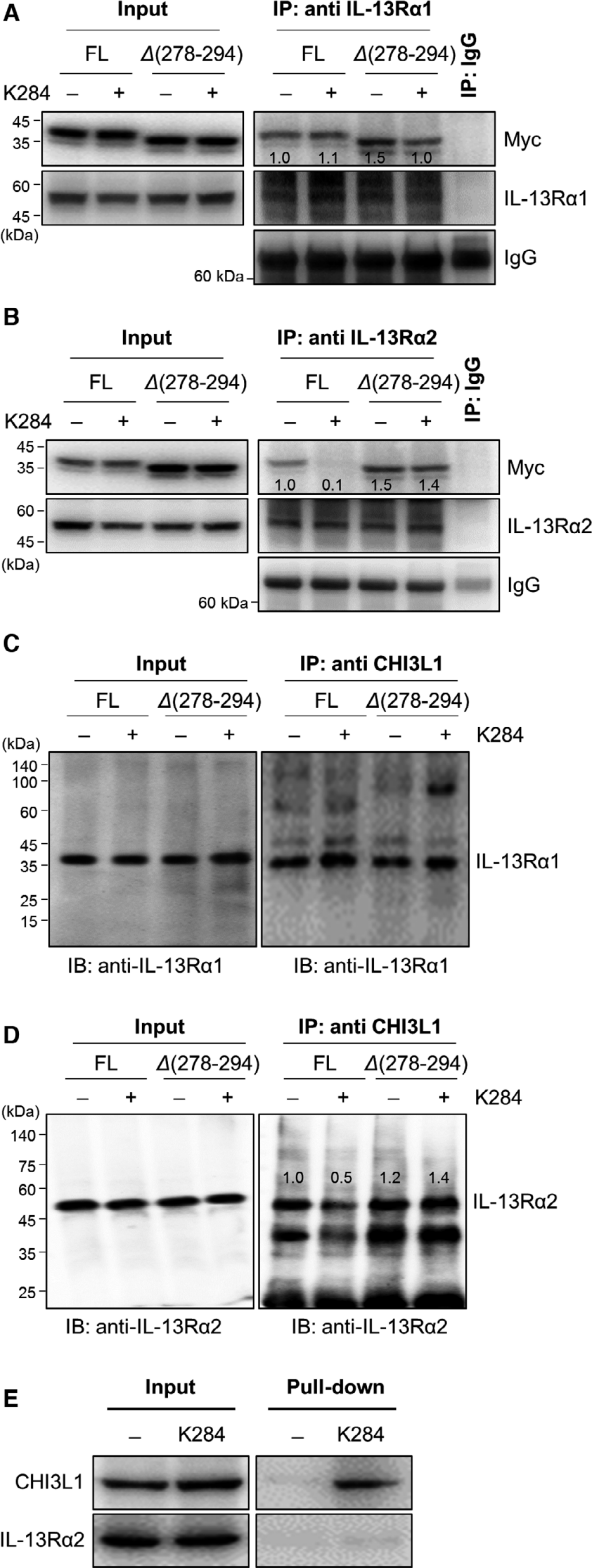
Effects of mutation of the docking site of CHI3L1 to K284 on the binding of IL‐13Rα2 receptor. A549 cells were transfected with FL or Δ278‐294 mutants of human CHI3L1‐6×Myc expression vector. Transfected cells were treated with or without 5 μm of K284 and incubated for 24 h. (A, B) Immunoprecipitation was performed with anti‐IL‐13Rα1 IgG or anti‐IL‐13Rα2 IgG. Blotting was performed with anti‐Myc IgG, and the bands showed Myc‐tagged CHI3L1. Input CHI3L1 (A, B; left) and immunoprecipitated CHI3L1 with IL‐13Rα1 (A; right) or IL‐13Rα2 (B; right) shown by western blotting. (C, D) Immunoprecipitation was performed with anti‐CHI3L1 IgG. Blotting was performed with anti‐IL‐13Rα1 IgG or anti‐IL‐13Rα2 IgG, and the bands showed IL‐13Rα1 (C) or IL‐13Rα2 (D). Input IL‐13Rα1 (C; left) or IL‐13Rα2 (D; left) and immunoprecipitated IL‐13Rα1 or IL‐13Rα2 with CHI3L1 (C, D; right) shown by western blotting. (E) Pull‐down assay showing the interaction among K284, human CHI3L1, and IL‐13Rα2. −; control unconjugated epoxy‐activated Sepharose 6B bead, +; K284 was conjugated with epoxy‐activated Sepharose 6B bead.

**Fig. 6:**
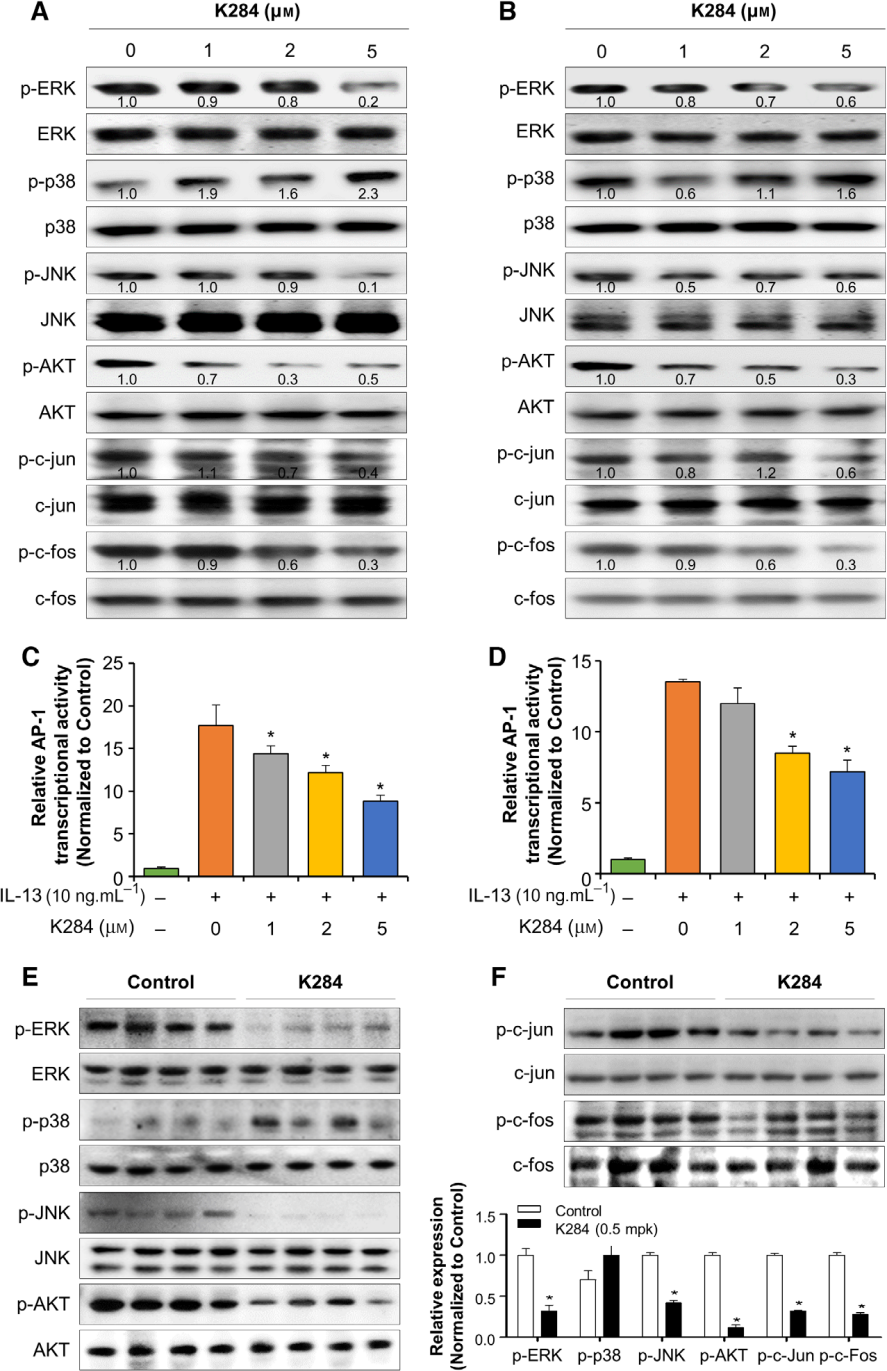
Inhibitory effect of K284 on the activation of JNK/AP‐1 signaling in A549 and H460 cell lines. (A, B) A549 (A) and H460 (B) cells were treated with 0–5 μm of K284 for 12 h. The expression of activated MAPK such as p‐ERK, p‐p38, p‐JNK, and p‐AKT and the nuclear components AP‐, p‐c‐Jun, and p‐c‐Fos from the total cell lysates was determined by western blotting. The values below the bands represent the band density. (C, D) AP‐1 consensus sequence containing the luciferase vector was transfected into A549 cells (C) or H460 cells (D). Cells were pretreated with K284 (0–5 μm) for 1 h and were then incubated with IL‐13 (10 ng·mL^−1^) for 6 h. AP‐1 transcriptional activity was measured by luminescence. *P < 0.05 (vs. control). (E, F) Western blot analysis for p‐ERK, ERK, p‐p38, p38, p‐JNK, JNK, p‐AKT, AKT, p‐c‐jun, c‐jun, p‐c‐fos, and c‐fos from the melanoma lung metastatic tissue lysate. The relative protein expression in the K284‐treated group compared with that in the control group is shown in the graphs. *P < 0.05 (vs. control). All error bars of graph reported are the standard deviation (SD) from three independent experiments.

**Fig. 7:**
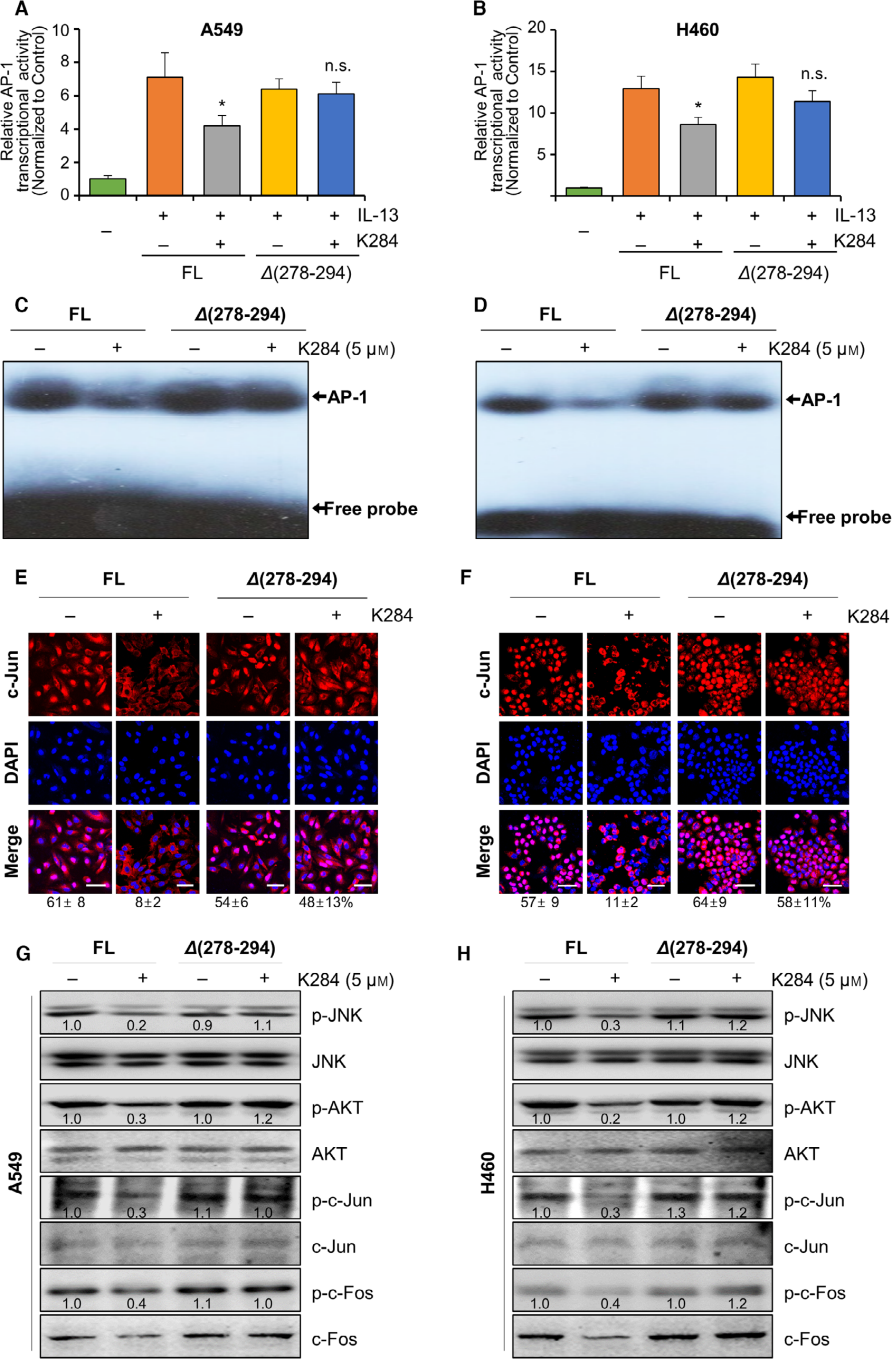
Abolished inhibitory effect of K284 on the activation of JNK/AP‐1 in mutant CHI3L1‐transfected A549 and H460 cells. (A, B) FL or Δ278‐294 mutants of human CHI3L1‐6× Myc‐expressing A549 cells and H460 cells were transfected with the AP‐1 luciferase vector for 6 h and then treated with IL‐13 with or without K284 for 24 h. Lane 1, empty vector‐expressing cells; lane 2, recombinant human IL‐13 (10 ng·mL^−1^)‐treated CHI3L1‐overexpressing cells; lane 3, recombinant human IL‐13 (10 ng·mL^−1^) and K284 (5 μm)‐treated CHI3L1‐overexpressing cells; lane 4, recombinant human IL‐13 (10 ng·mL^−1^)‐treated Δ278‐294 mutants of CHI3L1‐overexpressing cells; and lane 5, recombinant human IL‐13 (10 ng·mL^−1^) and K284 (5 μm)‐treated Δ278‐294 mutants of CHI3L1‐overexpressing cells. *P < 0.05 (vs. IL‐13 treatment). All error bars of graph reported are the standard deviation (SD) from three independent experiments. (C–H) FL or Δ278‐294 mutants of human CHI3L1‐6×Myc‐expressing A549 cells or H460 cells were treated with or without 5 μm K284. (C and D) Nuclear protein lysates were extracted and incubated together with AP‐1 consensus sequence labeled with γ‐^32^P‐ATP, and then, electrophoresis mobility shift assay was performed. (E and F) The translocation of c‐Jun from the cytosol to the nucleus was detected by confocal fluorescence microscopy. The percentage of c‐Jun‐positive cells were counted and shown below the images. Scale bar, 50 μm. (G and H) Western blot analysis for p‐JNK, JNK, p‐AKT, AKT, p‐c‐Jun, c‐Jun, p‐c‐Fos, and c‐Fos from A549 cells and H460 cells. Western blotting was repeated two times with duplicate samples. The values below the bands represent the band density.

GWAS/OMIM/DEG, BioMark, and GEO data analyses (Fig. S6A,B) and evidence from the literature have demonstrated that CHI3L1 shows its biological effects by binding to its receptor IL‐13Rαs. To address the inhibitory effect of K284 on the transduction of the CHI3L1/IL‐13Rαs signaling pathway, we investigated the interaction between CHI3L1 and IL‐13Rα1 or IL‐13Rα2 by immunoprecipitation after K284 treatment. A549 lung cancer cells were treated with K284 and then purified the cytosolic protein containing CHI3L1 and IL‐13Rαs to perform immunoprecipitation. We found that K284 inhibited the interaction between CHI3L1 and IL‐13Rα2, but the interaction between CHI3L1 and IL‐13Rα1 remained unaffected (Fig. 5A,B). K284 treatment that did not decrease the interaction of IL‐13Rα2 with CHI3L1 (Δ278‐294 mutation) protein inhibited the interaction between CHI3L1 FL protein and IL‐13Rα2 (Fig. 5A,B). This was confirmed using reversed immunoprecipitation assay (Fig. 5C,D). We further confirmed that K284 binds to CHI3L1 but not IL‐13Rα2 (Fig. 5E). These results showed that the CBD of CHI3L1 protein binds to IL‐13Rα2, and K284 interrupts this binding by specifically targeting CHI3L1. The suppressive effect of K284 on the CHI3L1/IL‐13Rα2 interaction could inhibit cancer cell growth‐related downstream signals. These results indicated that the downstream signals and cancer growth effects of CHI3L1 are suppressed by preventing the binding of CHI3L1 to its receptor IL‐13Rα2 by K284 treatment. We next studied the effects of K284 on CHI3L1/IL‐13Rα2 downstream pathways. Activator protein 1 (AP‐1) is a transcription factor of CHI3L1 and is activated by CHI3L1/IL‐13Rα2 signal transduction and related signaling molecules AKT and JNK [10]. We thus studied the AP‐1, AKT, and JNK signaling pathways and found that K284 significantly inhibits JNK activation among the MAPK pathway in a concentration‐dependent manner in A549 and H460 cell lines (Fig. 6A,B). Similarly, the expression of c‐Fos, c‐Jun, and AKT was also decreased in a concentration‐dependent manner (Fig. 6A,B). Because AP‐1, a complex of c‐Fos and c‐Jun, is a critical transcription factor for CHI3L1 expression and AKT pathway activation, we examined the transcriptional activity of AP‐1 after treatment with IL‐13, a ligand of IL‐13Rα2 that exhibits synergistic effects along with CHI3L1. K284 treatment decreased IL‐13‐induced AP‐1 transcriptional activity in a concentration‐dependent manner (Fig. 6C,D). To confirm these changes in lung tumor tissues, phosphorylation of these signals was detected in melanoma and lung cancer cell metastasis tumor tissues. Phosphorylation of ERK, JNK, and Akt was decreased in tumor tissues of K284‐treated mice (Figs 6E and S7A,B). Furthermore, the expression of p‐c‐Jun and p‐c‐Fos was also decreased by the K284 treatment (Figs 6F and S7A,B). Next, we performed the luciferase reporter assay to study the DNA‐binding activities of AP‐1 in either CHI3L1 FL or Δ278‐294 CHI3L1 expression vector‐transfected cells with/without K284 treatment. IL‐13‐induced AP‐1 transcriptional (Fig. 7A,B) and DNA‐binding activities (Fig. 7C,D) were significantly decreased by K284 treatment in CHI3L1 FL‐expressing cells. However, Δ278‐294 CHI3L1‐expressing cells showed no response to K284 treatment (Fig. 7A–D). Additionally, we investigated the transcriptional activities of other representative transcription factors, such as NF‐κB, SP‐1, and STAT3, after K284 treatment. The activities of SP‐1 and STAT3 induced by NF‐κB, USF1, and IL‐6 were not significantly affected by K284 treatment, but lipopolysaccharide‐ and TNF‐α‐induced SP‐1, STAT3, and AP‐1 were significantly decreased by K284 treatment in A549 lung cancer cells (Fig. S8). To confirm the nuclear translocation of c‐Jun, we investigated the immunofluorescence of K2841‐treated cells transfected with either FL or Δ278‐294 CHI3L1 expression vector. As shown in Fig. 7E,F, K284 treatment decreased the nuclear translocation of c‐Jun in CHI3L1 FL‐transfected but not in Δ278‐294 CHI3L1‐transfected A549 and H460 cells. To determine the effect of K284 on CHI3L1/IL‐13Rα2 downstream signal transduction, we investigated the phosphorylation of JNK and AKT after K284 treatment in CHI3L1 FL and Δ278‐294 CHI3L1‐transfected cells. The phosphorylation of JNK and AKT was decreased by K284 treatment in CHI3L1 FL‐transfected cells but not in Δ278‐294 CHI3L1‐transfected cells (Fig. 7G,H). The phosphorylation of c‐Fos and c‐Jun was also significantly reduced by K284 treatment in CHI3L1 FL‐transfected cells but not in Δ278‐294 CHI3L1‐transfected cells (Fig. 7G,H).

### 3.7 Expression of CHI3L1, IL‐13Rα2, and CHI3L1/IL‐13Rα2 downstream signals in the tumor tissues of lung cancer patients

We further investigated the involvement of CHI3L1 and IL‐3Rα2 in the development of lung tumors using tissues from patients. Our previous study demonstrated that the protein expression of CHI3L1 is elevated by the tumor stage‐dependent pattern in human lung tumor tissues [9]. Here, we investigated whether the protein expression of CHI3L1 correlates with IL‐13Rαs and its downstream signaling in tumor samples from lung cancer patients. As shown in Fig. S9, the protein expression of CHI3L1, IL‐13Rα1, and IL‐13Rα2 was significantly higher in tumor tissues than in normal tissues of lung cancer patients. Moreover, CHI3L1/IL‐13Rα2 downstream signaling was up‐regulated in the patient tissues (Fig. S9).

## 4 Discussion

Our previous analysis with GWAS/OMIM/DEG program confirmed the significant association between CHI3L1 and lung cancer development [9]. Dong *et al*. screened six candidate compounds from a library of 200 000 compounds using a structure‐based virtual screening approach and reported 2‐(3‐cyclopentylpropanamido)‐6‐methyl‐4,5,6,7‐tetrahydro‐benzo[*b*]thiophene‐3‐carboxylic acid propyl ester among those six candidate compounds to possess the strongest inhibitory activity [27]. However, this compound showed a binding affinity of about −8.76 to −6.96 kcal·mol^−1^ and it did not show any cancer cell‐killing effects. Our screening using the computational docking facility of Glide software yielded 11 compounds from a database of 1.4 million compounds at ChemBridge, of which K284 showed a strong affinity (*K_d_* = −9.7 kcal·mol^−1^) to CHI3L1. Our previous studies have shown that K284 have effects on neuroinflammatory disease and atopic dermatitis. In this study, we investigated the anticancer effect of CHI3L1‐inhibiting chemicals [28, 29, 30].

Therapy techniques for lung cancer with high incidence and mortality rates continue to develop. Several studies demonstrated that high levels of CHI3L1 were expressed in lung cancer patient tissue and serum, and lung metastasis was decreased in Chi3L1 depleted mice [19, 22, 31]. K284 significantly inhibited lung cancer cell growth at 5 μm (IC_50_ = 2 μm). As the tumor grows, they can invade the surrounding tissue and enter the lymphatic tract, and where some cancer cells break away from the primary tumor and enter the lymphatic tract, the cell enters the lymphatic gland and then settles in the surrounding lymph nodes. These metastases appear in malignant solid tumors and are often found in malignant melanoma. The tail vein injection is a well‐established metastatic model that confirms tumor growth in metastatic tissue sites. In our B16F10 metastasis model, K284 was shown to significantly inhibit the number of metastatic nodules at a dose of only 0.5 mg·kg^−1^. Moreover, another metastatic model, the A549 cell‐injected model, also showed that K284 inhibited lung metastasis. Two different models of tumor metastasis provide similar results in which K284 treatment showed antimetastatic effects. Additionally, K284 significantly and consistently decreased the expression of cancer growth‐, migration‐, and cell death‐associated proteins, but increased Bax and cleaved caspase‐3 levels in A549 and H460 human lung cancer cells and three lung tumor model tissues. Thus, these results indicate that K284 significantly inhibits lung tumor metastases by targeting CHI3L1 [31, 32]. However, the underlying mechanisms by which K284 acts on CHI3L1 and CHI3L1‐associated signaling in the metastases of lung cancer could not be completely elucidated.

The catalytic domain and the CBD of CHI3L1 (between 22 and 357‐AA) are required to bind to full‐length IL‐13Rα2 [10]. Interestingly, the CBD is necessary for CHI3L1/IL‐13Rα2 binding. Our docking model and pull‐down assay demonstrated that K284 directly binds to CBD region of CHI3L1. In addition, K284 inhibited the interaction between CHI3L1 and IL‐13Rα2. These results indicate that the binding of K284 to the CBD (phenylalanine 287, threonine 288, lysine 289, glutamate 290, and threonine 293) of human CHI3L1 could block the interaction of CHI3L1 with IL‐13Rα2. However, we did not observe any inhibitory effect of K284 on the interaction between CHI3L1 and IL‐13Rα1, suggesting that the inhibitory effect of K284 is specific to IL‐13Rα2. CHI3L1 specifically binds to IL‐13Rα2 in cancer cells, thus promoting cancer growth [33]. Even though the binding site of CHI3L1 to CD44 is not clear, it is known that CHI3L1 can bind to CD44 and cause gastric cancer cell metastasis [31]. The interaction between CHI3L1 and IL‐13Rα2 could promote lung diseases associated with Hermansky–Pudlak syndrome [34]. He *et al*. [10] reported that CHI3L1 binds to IL‐13Rα2 and activates IL‐13Rα2‐dependent MAPK, AKT, and Wnt/β‐catenin signaling pathways, thereby regulating apoptosis, inflammasome activation, and melanoma metastasis. The biological significance of the interaction between CHI3L1 and IL‐13Rα2 has been demonstrated. Gene–disease and gene–gene network analyses indicated that CHI3L1 and IL‐13Rα2 are commonly associated with many diseases including lung cancer and these genes are colocalized. Pep‐1L, an IL‐13Rα2 targeting peptide, reduces glioblastoma development [35]. The inhibitory effects of K284 on cancer cell growth, cell cycle arrest and apoptosis, and the expression of related proteins were not observed in CHI3L1 cells that had a deletion mutation for the binding region. Thus, it can be speculated that inhibition of the interaction between CHI3L1 and IL‐13Rα2 by K284 could prevent lung cancer cell metastasis and growth.

Concerning the changes in the downstream signaling pathways induced by inhibition of CHI3L1/IL‐13Rα2 signaling, the interaction of CHI3L1/IL‐13Rα2 was reported to trigger the phosphorylation of JNK. The phosphorylation of JNK led to the activation of AP‐1 transcription factors in the nuclei [13, 36] and thus promoted the AP‐1‐dependent expression of target genes related to cell growth, metastasis, and cell death [13, 35, 36, 37]. CHI3L1 activates the IL‐13Rα2‐dependent activation of the AKT pathway in melanoma metastasis [10]. The CHI3L1/IL‐13Rα2 complex accompanied by TMEM219 promotes oxidant‐induced apoptosis, lung injury, and melanoma metastasis through activation of the macrophage ERK/AKT pathway [30]. In our *in vivo* study, consistent with the inhibitory effects of K284 on tumor metastasis, JNK, ERK, and AKT levels, as well as c‐Jun and c‐Fos phosphorylation, were significantly decreased by K284. In our *in vitro* study, inhibitory effects of K284 on the binding of CHI3L1 to IL‐13Rα2 blocked JNK, AKT, and AP‐1 signaling and the expression of related genes (c‐Fos and c‐Jun) in cultured lung cancer cells. Moreover, in cells containing a deletion mutation in the CBD of CHI3L1, the inhibitory effects of K284 on the IL‐13Rα2‐mediated JNK, AKT, and AP‐1 signaling, as well as cell growth, migration, and apoptotic cell death, were abolished. Thus, our data indicated that K284 blocks the formation of the CHI3L1/IL‐13Rα2 complex and the downstream signaling of JNK, AKT, and AP‐1 pathways leading to the inhibition of cancer cell growth. Several transcription factors, such as USF1, STAT3, NF‐κB, SP‐1, and AP‐1 contribute to CHI3L1 expression. In the present study, K284 inhibited lipopolysaccharide‐, TNF‐α, and IL‐6‐induced transcriptional activation of AP‐1 but not SP‐1 and STAT3. These data suggested that the CHI3L1/IL‐13Rα2 signal‐dependent AP‐1 pathway might play a significant role in the direct inhibitory effects of K284 on cancer cell growth. Interestingly, we found that the level of IL‐13Rα2 expression was not changed, indicating that the binding of CHI3L1 to IL‐13Rα2 may be sufficient to transduce the signals to downstream pathways. In the tissue samples from lung cancer patients, the protein expression of CHI3L1, IL‐13Rα1, and IL‐13Rα2 and the phosphorylated levels of c‐Jun, c‐Fos, AKT, and ERK were elevated compared with those in normal lung tissues. Therefore, the binding of K284 to CHI3L1 could block CHI3L1/IL‐13Rα2 complex formation and inhibit lung tumor growth. K284 is predicted to exhibit drug‐like properties as evaluated by *in* *silico* absorption, distribution, metabolism, excretion, and toxicology studies (data not shown).

## 5 Conclusion

This study demonstrated that K284 is a potential anticancer drug candidate that targets CHI3L1 to block IL‐13Rα2‐mediated JNK/AP‐1 signaling.

## Conflict of interest

The authors declare no conflict of interest.

## Author contribution

YSL and JEY conducted most of the experiments, performed data analysis, and were the primary writer of the manuscript. KCK assisted the in vivo and in vitro experiments. DHL, DJS, and HPL assisted data interpretation. JKJ, NDK, and YWH designed and synthesized testing compounds. JJ and SBH provided advice throughout the project. JTH supervised the entire project and had a major role in experimental design, data interpretation, and writing of the manuscript. All authors reviewed the manuscript.

### Peer review

The peer review history for this article is available at https://publons.com/publon/10.1002/1878‐0261.13138.

## Supporting information


**Fig. S1**. Structure and docking score of selected chemical compounds.
**Fig. S2**. Anti‐cancer effect of selected chemical compounds.
**Fig. S3**. Anti‐cancer effect of K284 in lung cancer cells.
**Fig. S4**. Effect of K284 on the expression of cell growth, migration, and apoptosis related proteins.
**Fig. S5**. Mutation effects of docking site of CHI3L1 to K284 on cell growth and migration.
**Fig. S6**. CHI3L1 and IL‐13Rα2 gene/disease network.
**Fig. S7**. Inhibitory effect of K284 on the activation of JNK/AP‐1 signals in A549 lung metastasis model and melanoma tumor growth model.
**Fig. S8**. Transcriptional activity of CHI3L1‐related transcription factors.
**Fig. S9**. Expression of CHI3L1, IL‐13Rα2, Chi3L1/IL‐13Rα2 downstream signals in the lung tumor patient tissues.

## Data Availability

No data sets were generated or analyzed during this study.
