# Supplementary material for: A small molecule targeting CHI3L1 inhibits lung metastasis by blocking IL‐13Rα2‐mediated JNK‐AP‐1 signals
Source: Mol Oncol. 2021 Nov 24;16(2):508–26. doi: 10.1002/1878-0261.13138 (PMC8763653; doi:10.1002/1878-0261.13138)
Supplement: Supplementary file 1 — Fig. S1. Structure and docking score of selected chemical compounds. Fig. S2. Anti‐cancer effect of selected chemical compounds. Fig. S3. Anti‐cancer effect of K284 in lung cancer cells. Fig. S4. Effect of K284 on the expression of cell growth, migration, and apoptosis related proteins. Fig. S5. Mutation effects of docking site of CHI3L1 to K284 on cell growth and migration. Fig. S6. CHI3L1 and IL‐13Rα2 gene/disease network. Fig. S7. Inhibitory effect of K284 on the activation of JNK/AP‐1 signals in A549 lung metastasis model and melanoma tumor growth model. Fig. S8. Transcriptional activity of CHI3L1‐related transcription factors. Fig. S9. Expression of CHI3L1, IL‐13Rα2, Chi3L1/IL‐13Rα2 downstream signals in the lung tumor patient tissues. [file MOL2-16-508-s001.docx]

**Supporting information**

A small molecule targeting CHI3L1 inhibits lung metastasis by blocking IL-13Rα2-mediated JNK-AP-1 signals

Yong Sun Lee^1*^, Ji Eun Yu^1*^, Ki Cheon Kim^1^, Dong Hun Lee^1^, Dong Ju Son^1^, Hee Pom Lee^1^, Jae-kyung Jung^1^, Nam Du Kim^2^, Young Wan Ham^3^, Jaesuk Yun^1^, Sang-Bae Han^1^ and Jin Tae Hong^1#^

^1^College of Pharmacy & Medical Research Center, Chungbuk National University, Osongsaengmyeong 1-ro 194-21, Osong-eup, Heungduk-gu, Cheongju, Chungbuk, 28160, Republic of Korea,

^2^Voronoibio Inc. Songdo-Technopart IT Center 32 Songdogwahak-ro, Yeonsu-gu, Incheon, 21982, Republic of Korea

^3^Department of Chemistry, Utah Valley University 800 W. University Pkwy. Orem, UT 84058, USA

^*^: These authors contributed equally to this work.

**^#^Corresponding author:** Dr. Jin Tae Hong (jinthong@chungbuk.ac.kr), College of Pharmacy and Medical Research Center, Chungbuk National University, 194-21 Osongsaengmyeong 1-ro, Osong-Biocampus, Osong-eup, Heungdeok-gu, Cheongju, Chungbuk 28160, Korea. Phone: +82-43-261-2813; FAX: +82-43-268-2732.

**Supplementary Figure legends**

**
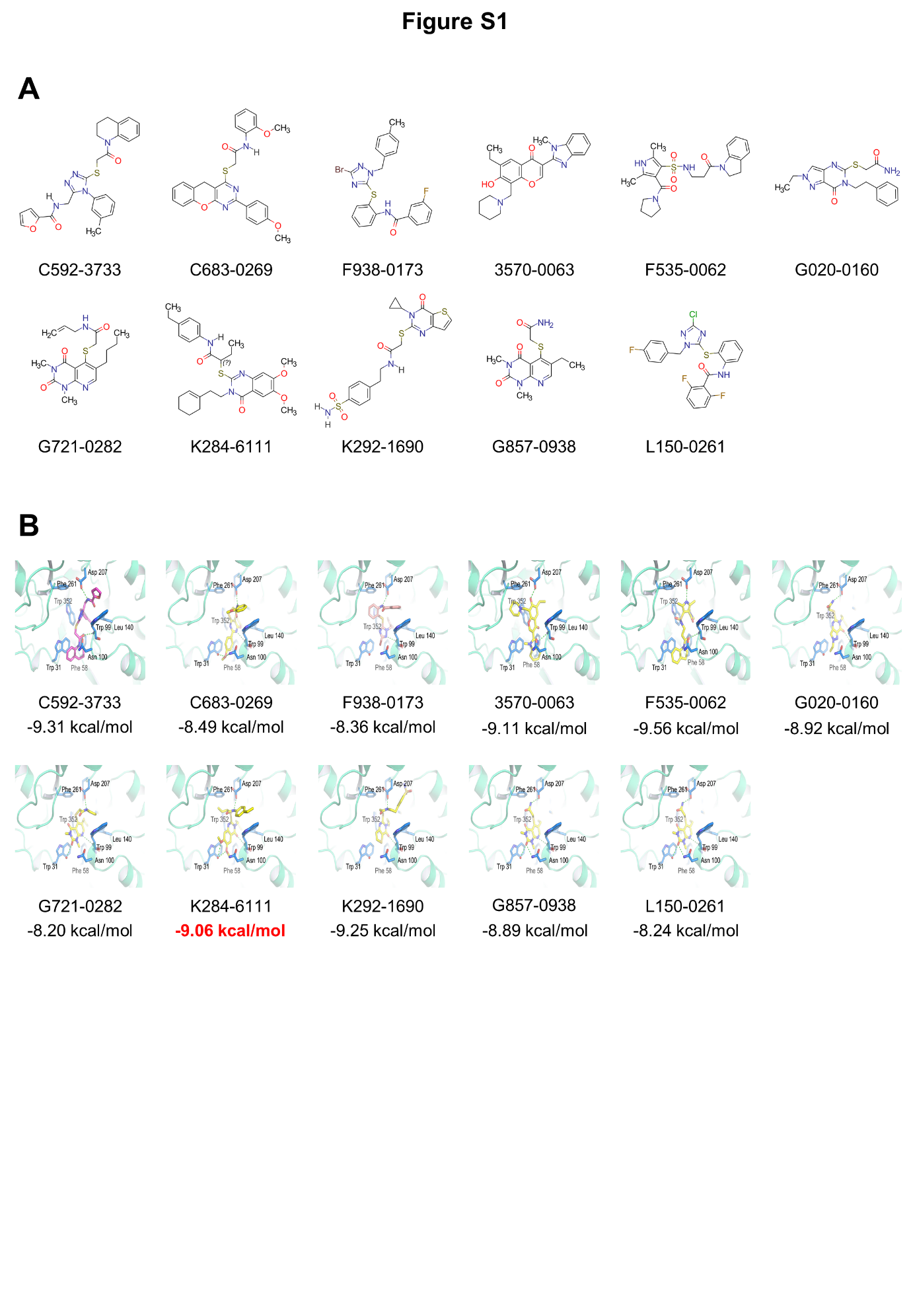
**

**Supplementary Figure S1. Structure and docking score of selected chemical compounds.**

(A) 11 compounds predicted to bind to human Chi3L1 from Chembridge library. (B) Chi3L1-chemcal docking models using Glide software. The code and docking score (kcal/mol) for each compound were shown under each compound.

**
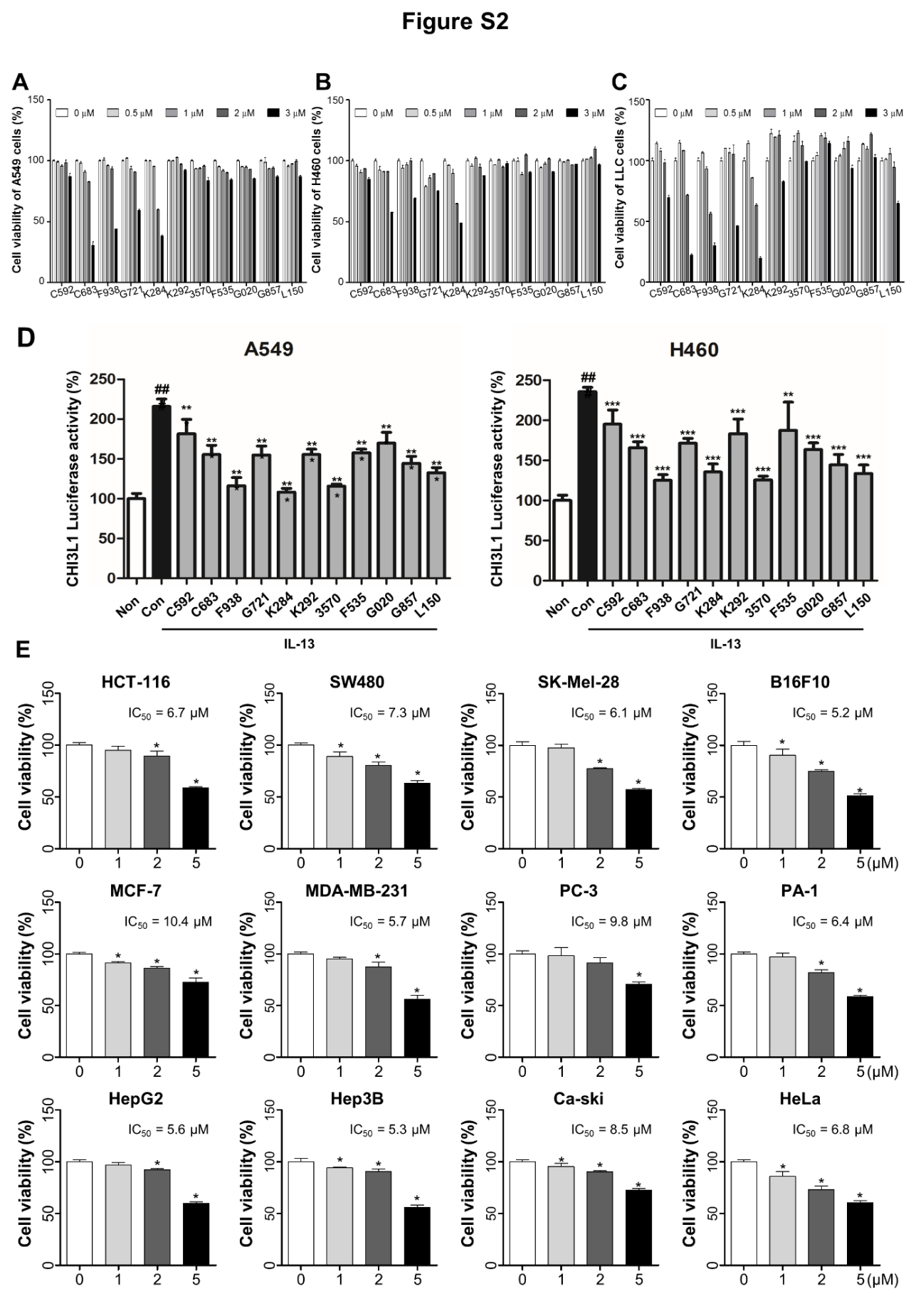
**

**Supplementary Figure S2. Anti-cancer effect of selected chemical compounds.**

(A-C) A549, H460, and LLC cell lines were treated with 11 chemical compounds, and cell growth determined with MTT assay. (D) A549 and H460 cells were treated pretreated with 5 μM of K284 for 1 h, and then were incubated with LPS (50 ng/mL) for 6 h. Transcriptional activities were measured by luminescence. Values are from three experiment with duplicates. **, *P* < 0.01 (vs. Control); ***, *P* < 0.001 (vs. Control). (E) K284 treatment at different concentration to various cancer cell lines. **P* < 0.05 (vs. Control). All error bars of graph reported are the standard deviation (SD) from three independent experiments.

**
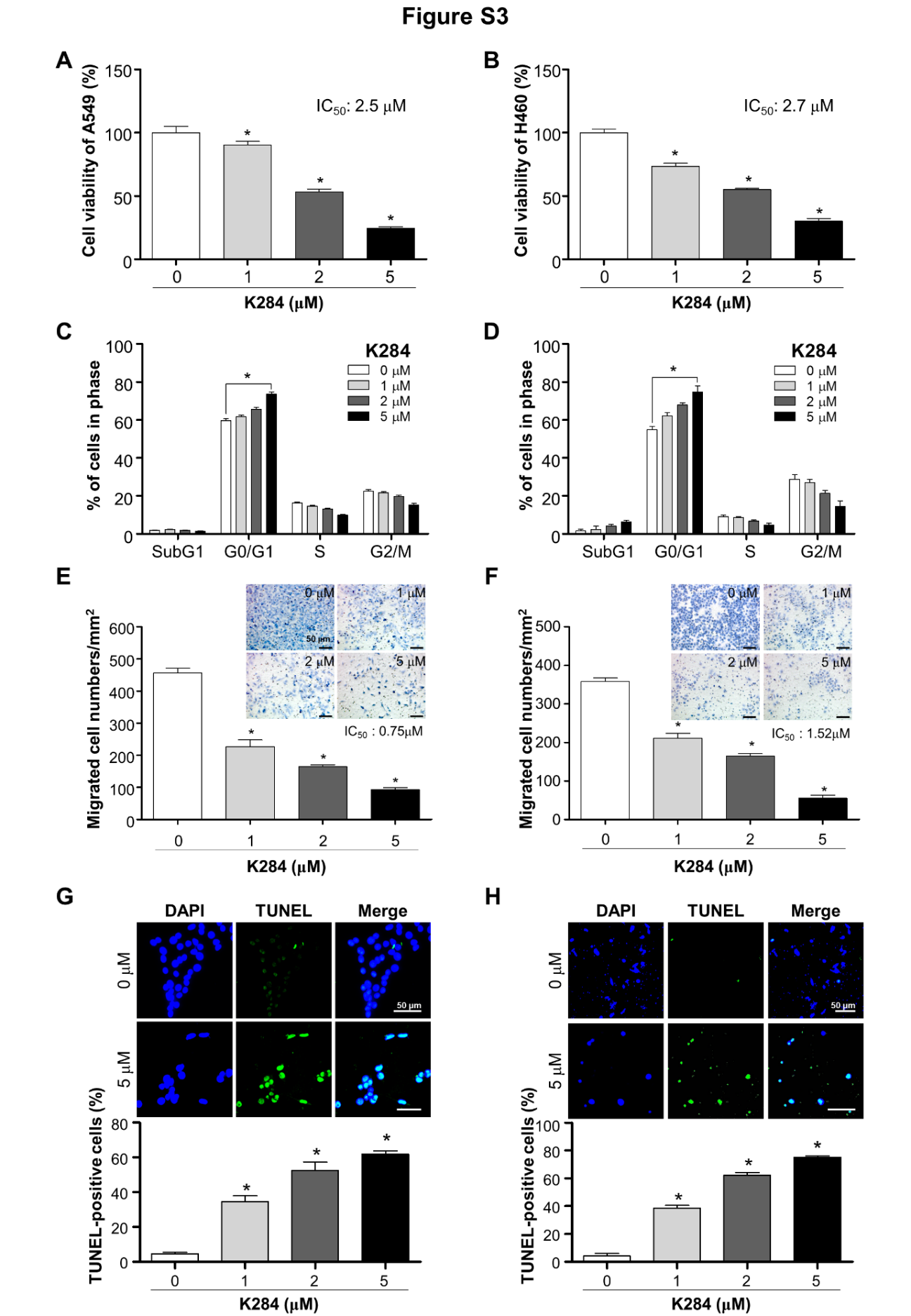
**

**Supplementary Figure S3.  Anti-cancer effect of K284 in lung cancer cells.**

A549 cells and H460 cells were treated with 0 to 5 μM of K284 for 48 h (A, B, E, F, G and H) or 24 h (C and D). (A-B) Cell viability was determined by MTT assay. (C-D) Cell cycle analysis was performed by flow cytometer. (E-F) Cell migration was determined by trans-well migration assays. Migrated cell numbers showed in lower panel. (G-H) Apoptosis was determined with TUNEL assay. Data are expressed as the mean ± S.E.M. of three experiments. Scale bar, 50 μm. **P* < 0.05 vs. control.


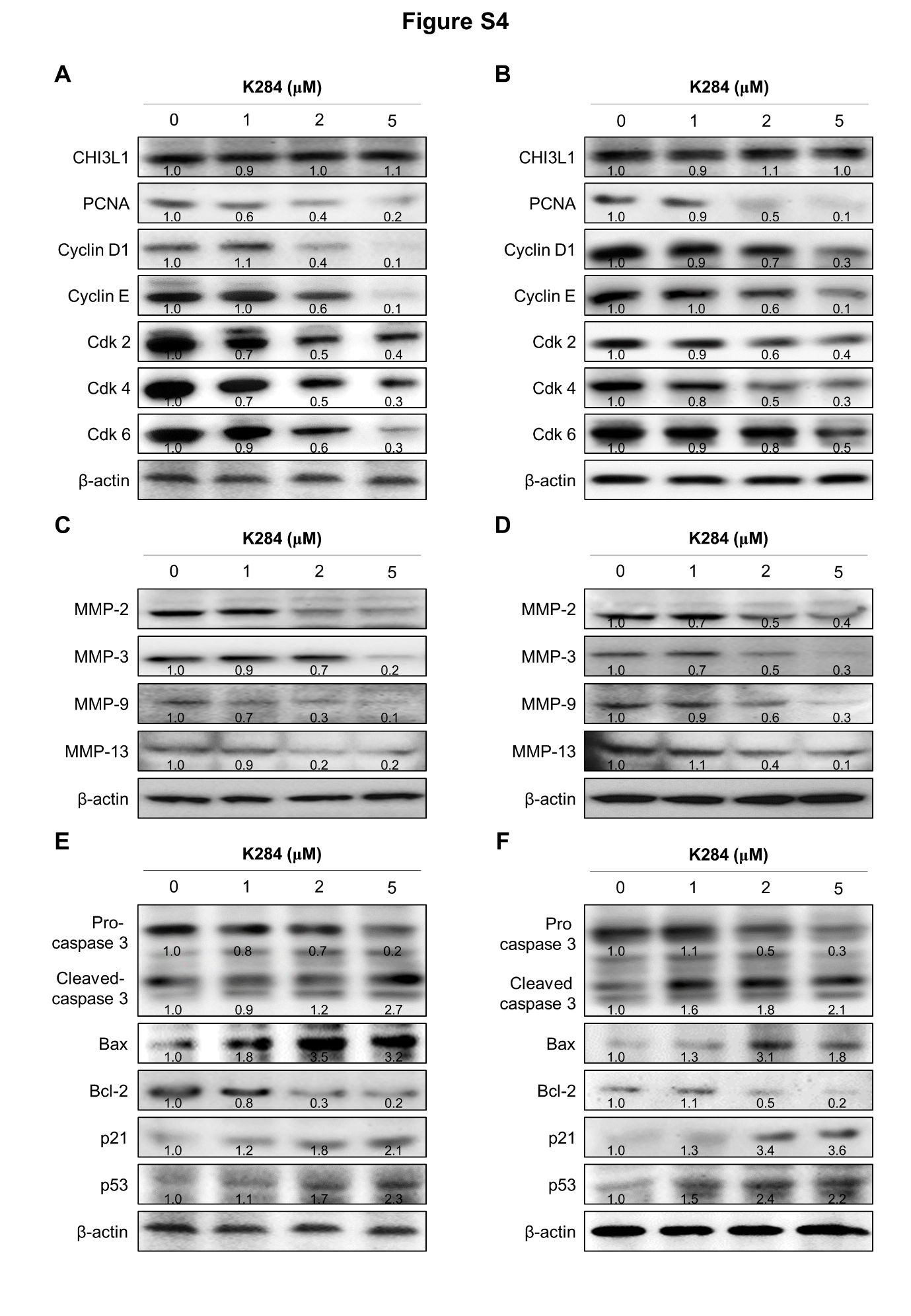


**Supplementary Figure S4. Effect of K284 on the expression of cell growth, migration, and apoptosis related proteins.**

A549 cells and H460 were treated with 0 to 5 μM of K284 for 24 h. (A-B) The expression of cell cycle related proteins such as CDK2, CDK4, CDK6, cyclin D1, and cyclin E as well as PCNA for cell proliferation marker was measured by Western blotting. (C-D) The expression of cell migration related proteins such as MMP2, MMP3, MMP9, and MMP13 was measured by Western blotting. (E-F) The expression of apoptotic cell death related proteins such as caspase 3, Bax, Bcl-2, p21, and p53 was measured by Western blotting in A549 and H460. Values under bands indicate related density. The experiments were repeated with duplicates.


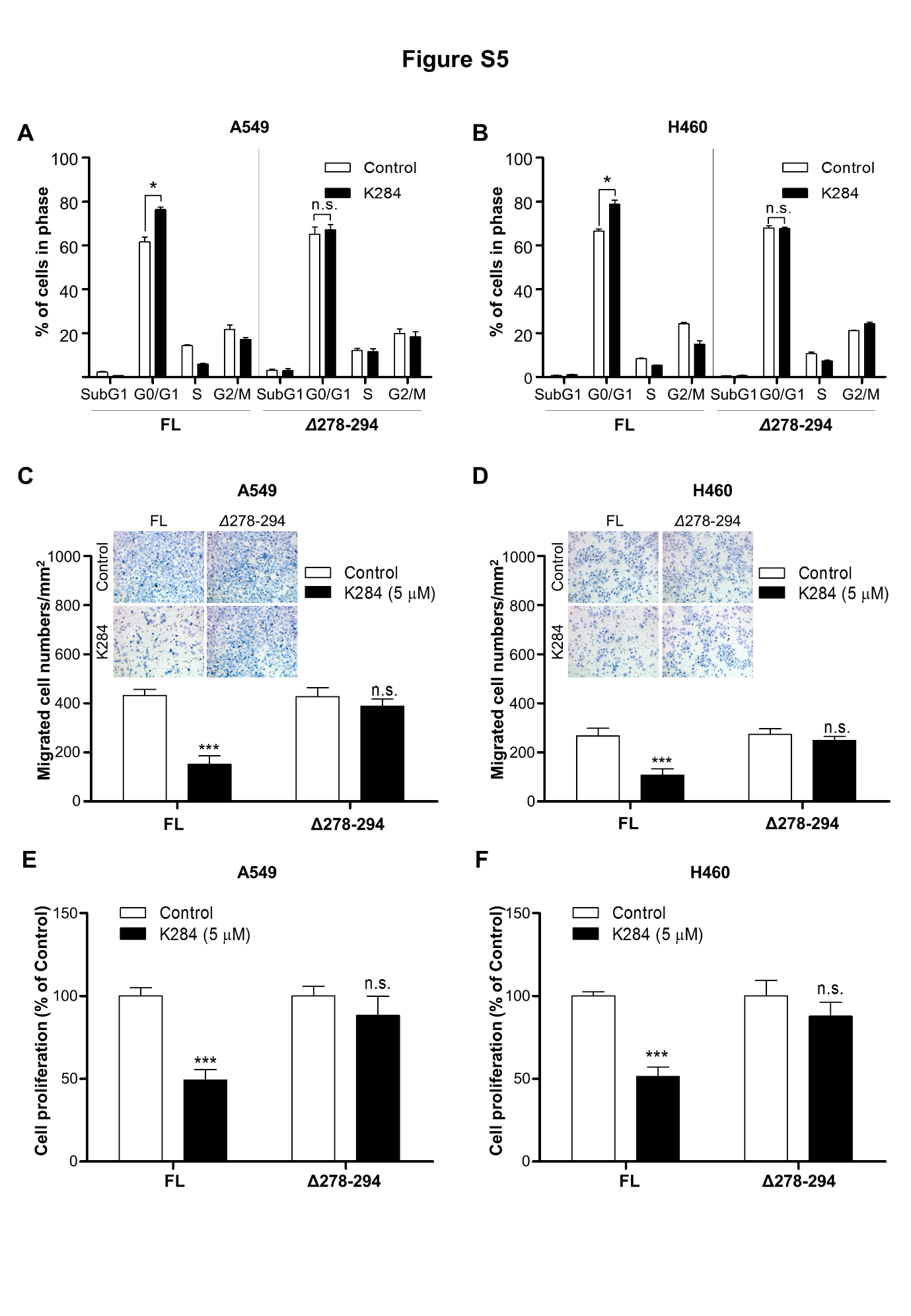


**Supplementary Figure S5.  Mutation effects of docking site of CHI3L1 to K284 on cell growth and migration.**

(A-B) Full length or 278-294-AA-deleted mutation of human Chi3L1-6×Myc expressed A549 (A) and H460 cells (B) were treated with 5 μM of K284. Cell cycle was measured after 12 h treatment of K284. **P* < 0.05 vs. Control. (C-F) Full length or 278-294-AA deleted mutation of human Chi3L1-6×Myc expressed A549 cells and H460 cells were treated with 5 μM of K284 for 24 h. The migrated cell numbers showed in lower panel (C-D). ****P* < 0.001 vs. control. Cell viability was determined by MTT assay (E-F). Data are expressed as the mean ± SD of three experiments. ****P* < 0.001 vs. Control


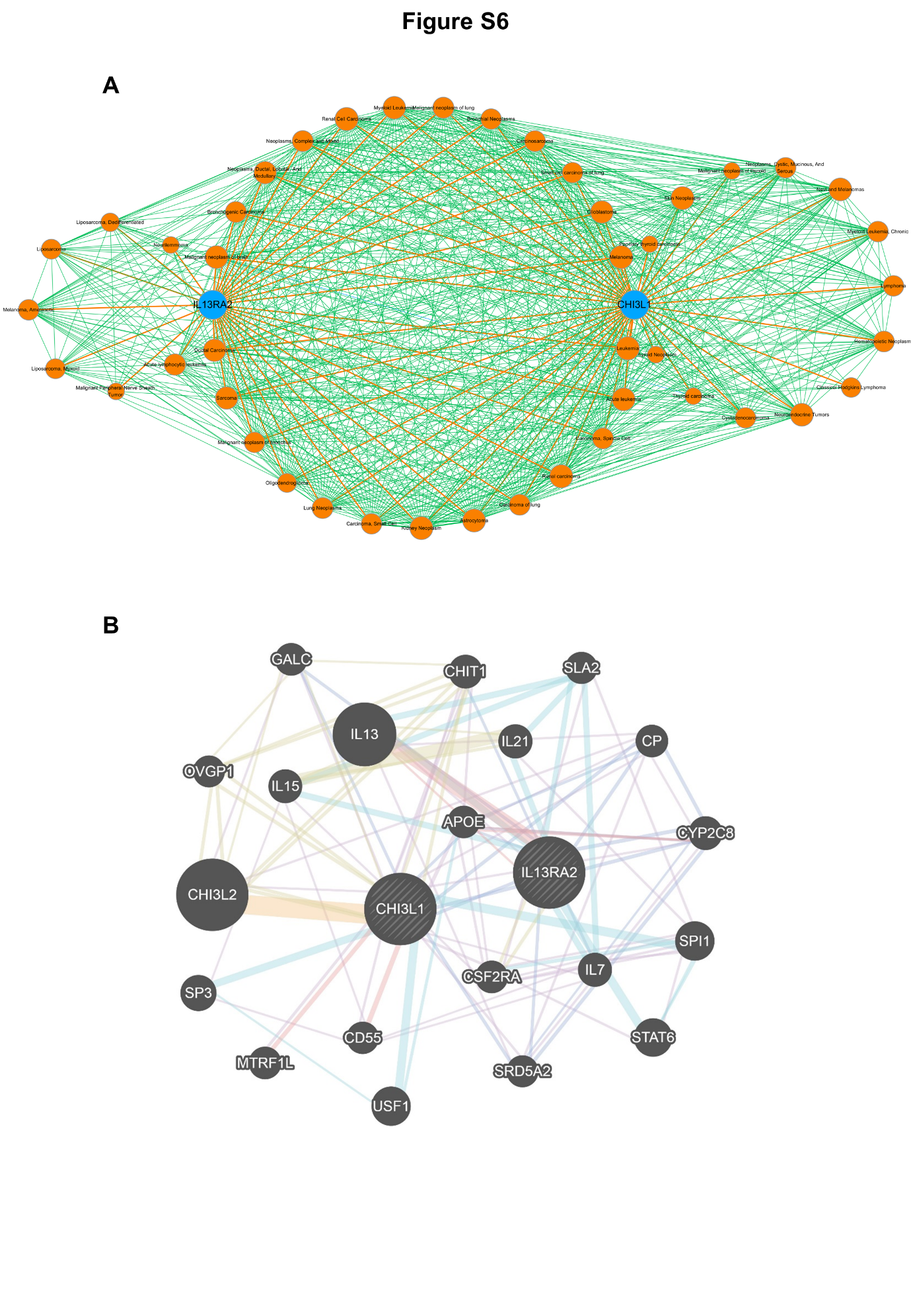


**Supplementary Figure S6. CHI3L1 and IL-13Rα2 gene/disease network.**

(A) Relationship between IL-13Rα2 and Chi3L1 on various disease was predicted by GWAS/OMIN/DEG database. (B) Genetic relationship between Chi3L1 and IL-13Rα2 was predicted by Biomart and GEO analysis.


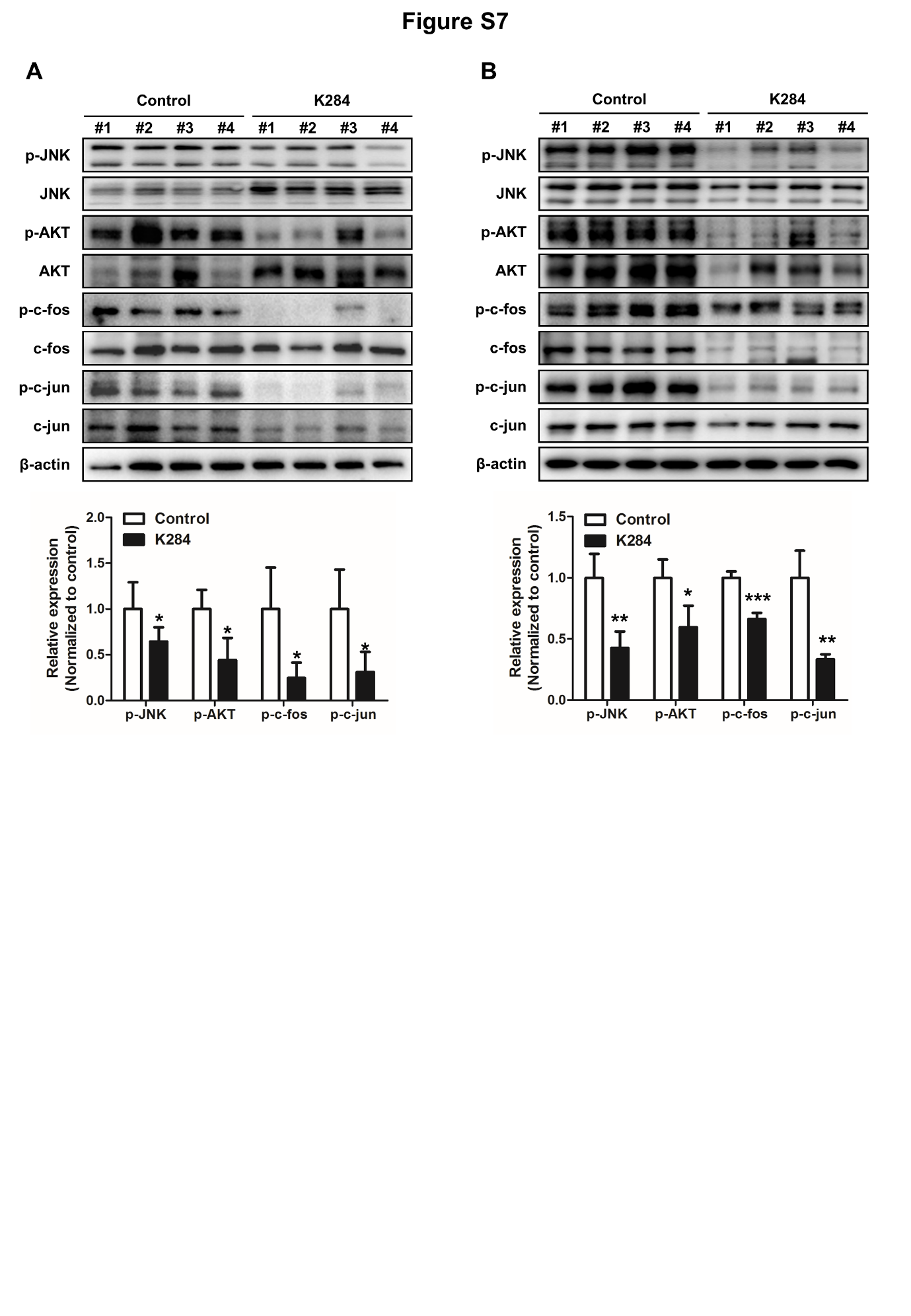


**Supplementary Figure S7.  Inhibitory effect of K284 on the activation of JNK/AP-1 signals in A549 lung metastasis model and melanoma tumor growth model.**

(A) B16F10 melanoma cells were injected in C57BL/6 mice that were then intravenously administered via the tail vein saline (left) or 0.5 mg/kg K284 (right) at 3-day intervals for 3 weeks. Western blot analysis for p-JNK, JNK, p-AKT, AKT, p-c-fos, c-fos, p-c-jun, c-jun from the lung tumor metastatic tissue lysate of C57BL/6 mice. The relative protein expression in the K284-treated group compared with that in the control group is shown in the graphs. **P* < 0.05 vs. control. (B) A549 human lung cancer cells were injected in BALB/c nude mice that were then intravenously administered via the tail vein saline (left) or 0.5 mg/kg K284 (right) at 3-day intervals for 8 weeks. Western blot analysis for for p-JNK, JNK, p-AKT, AKT, p-c-fos, c-fos, p-c-jun, c-jun from the lung tumor metastatic tissue lysate. The relative protein expression in the K284-treated group compared with that in the control group is shown in the graphs. **P* < (0.05 vs. Control); **, *P* < 0.01 (vs. Control); ***, *P* < 0.001 (vs. Control).


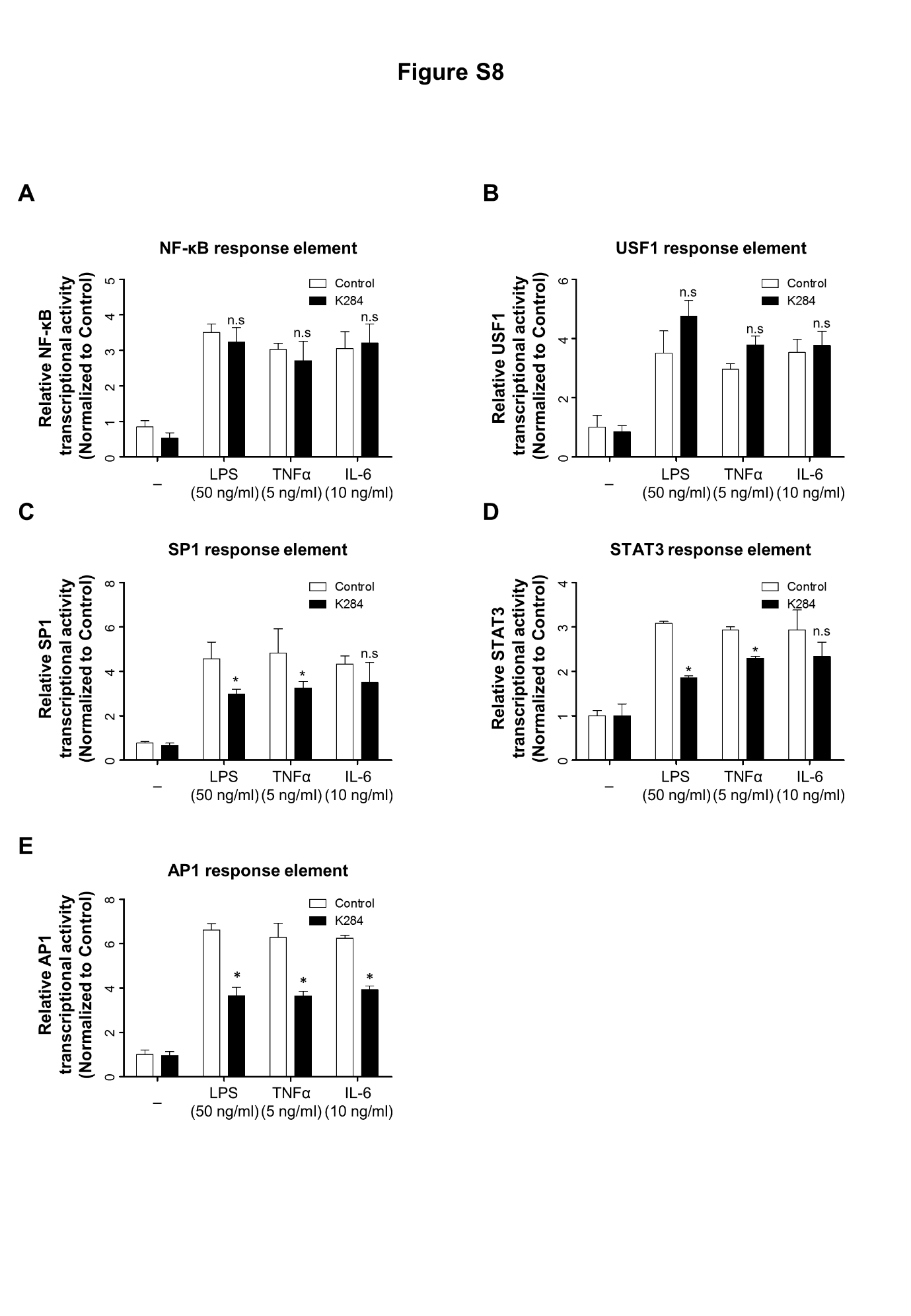


**Supplementary Figure S8. Transcriptional activity of CHI3L1-related transcription factors.**

(A-E) NF-κB, USF1, SP-1, STAT3 and AP1 consensus sequence contained luciferase vector were transfected to A549 cell line. Cells were pretreated with 5 μM of K284 for 1 h, and then were incubated with LPS (50 ng/mL), TNF-α (5 ng/mL) and IL-6 (10 ng/mL) for 6 h. Transcriptional activities were measured by luminescence. Values are from three experiment with duplicates. **P* < 0.05 vs. control.


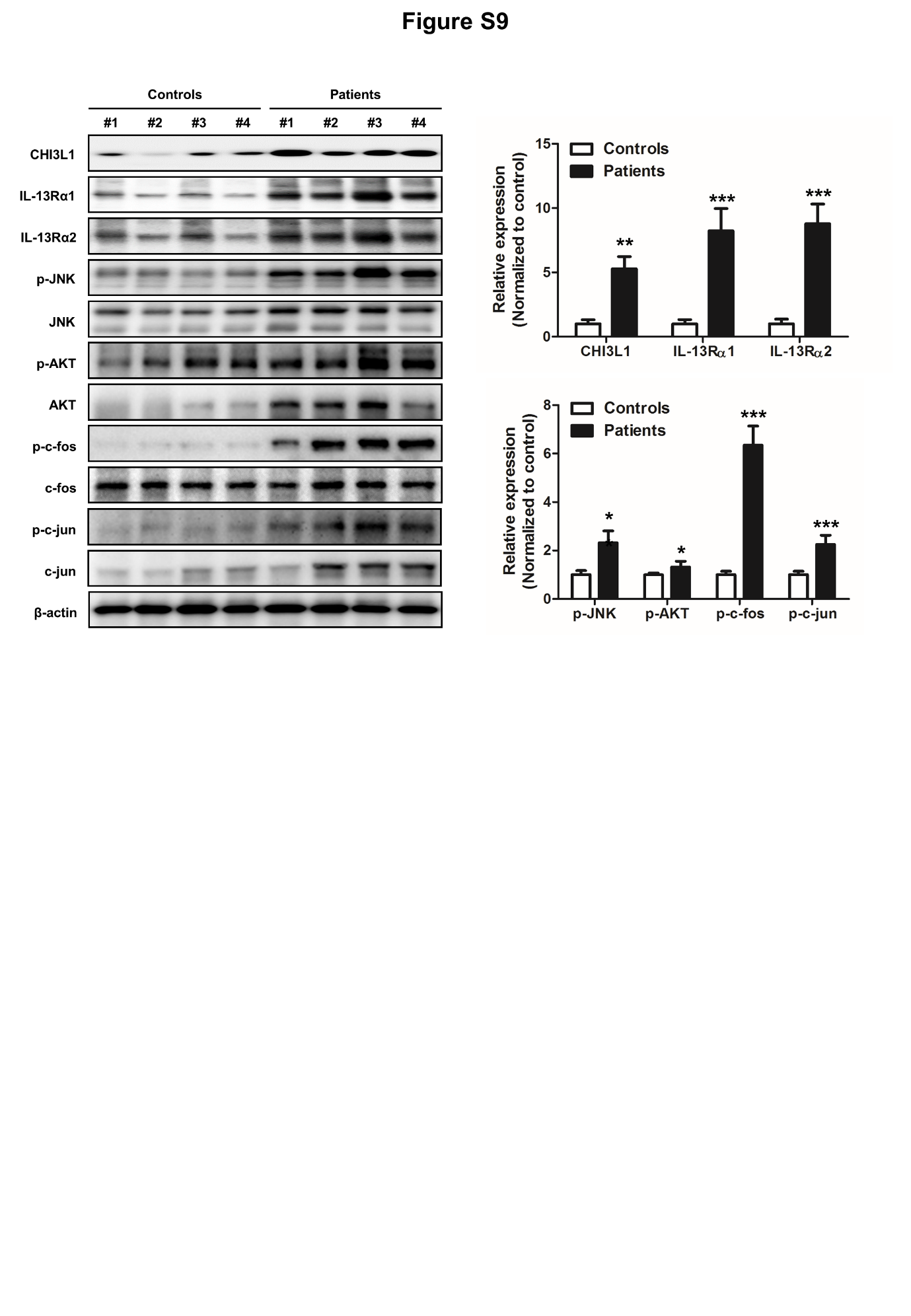


**Supplementary Figure S9. Expression of CHI3L1, IL-13Rα2, Chi3L1/IL-13Rα2 downstream signals in the lung tumor patient tissues.**

(A) The expression of CHI3L1, IL-13Rα1, IL-13Rα2, p-JNK, JNK, p-AKT, AKT, p-c-foc, c-fos, p-c-jun, and c-jun from human patients tissue cell lysates was determined by Western blotting. The relative protein expression in the K284-treated group compared with that in the control group is shown in the graphs. **P* < 0.05 (vs. Control.); **, *P* < 0.01 (vs. Control); ***, *P* < 0.001 (vs. Control).
